# Lifespan Dispersion in Times of Life Expectancy Fluctuation: The Case of Central and Eastern Europe

**DOI:** 10.1007/s13524-018-0729-9

**Published:** 2018-11-12

**Authors:** José Manuel Aburto, Alyson van Raalte

**Affiliations:** 1 Interdisciplinary Center on Population Dynamics, University of Southern Denmark, Odense, Denmark; 2 Max Planck Institute for Demographic Research, Rostock, Germany

**Keywords:** Causes of death, Alcohol consumption, Mortality, Health inequalities, Decomposition techniques

## Abstract

Central and Eastern Europe (CEE) have experienced considerable instability in mortality since the 1960s. Long periods of stagnating life expectancy were followed by rapid increases in life expectancy and, in some cases, even more rapid declines, before more recent periods of improvement. These trends have been well documented, but to date, no study has comprehensively explored trends in lifespan variation. We improved such analyses by incorporating life disparity as a health indicator alongside life expectancy, examining trends since the 1960s for 12 countries from the region. Generally, life disparity was high and fluctuated strongly over the period. For nearly 30 of these years, life expectancy and life disparity varied independently of each other, largely because mortality trends ran in opposite directions over different ages. Furthermore, we quantified the impact of large classes of diseases on life disparity trends since 1994 using a newly harmonized cause-of-death time series for eight countries in the region. Mortality patterns in CEE countries were heterogeneous and ran counter to the common patterns observed in most developed countries. They contribute to the discussion about life expectancy disparity by showing that expansion/compression levels do not necessarily mean lower/higher life expectancy or mortality deterioration/improvements.

**Electronic supplementary material:**

The online version of this article (10.1007/s13524-018-0729-9) contains supplementary material, which is available to authorized users.

## Introduction

The twentieth century was marked by sizable improvements in mortality and health in most countries in the world (World Health Organization 2000). However, these improvements were unevenly shared in the second half of the past century: parts of Central and Eastern Europe (CEE) experienced an unprecedented period of stagnation and, in some countries, decreases in life expectancy at birth around the mid-1960s (Human Mortality Database 2016). The long-term combination of a failure to complete the epidemiologic transition by reducing cardiovascular diseases (Caselli et al. 2002) and fluctuation in alcohol consumption and violence, particularly in the countries of the former Soviet Union (FSU) (Bye 2008; Leon et al. 1997), led to lower levels of life expectancy and larger within-country mortality inequalities according to education level in this region compared with countries in Western Europe (Mackenbach et al. 2008). The high mortality among young men is at the heart of the unstable Eastern European trends in life expectancy (McKee and Shkolnikov 2001). For example, male life expectancy stagnated at a level between 65 and 70 years from the 1960s to the mid-1980s in most countries of the region. Russia experienced the lowest male life expectancy in the region over this period, which was followed by a brief period of sizable improvements in life expectancy due to Gorbachev’s anti-alcohol campaign (Leon and Shkolnikov 1998). After 1987, the mortality experiences in the region diverged. Life expectancy increased continuously in parts of Central Europe. The remaining countries, particularly those from the FSU, experienced a pronounced period of deterioration up to the mid-1990s. Mortality increases among Russian and Latvian men were especially sharp, with life expectancy losses of approximately 7.5 years between 1987 and 1994, which led to levels not seen since the 1950s (Shkolnikov et al. 2001). Since the mid-1990s, life expectancy has mostly been increasing throughout the region but at different rates. As a result, large regional differences in survival have emerged. For instance, the 2010 gap in male life expectancy between Slovenia and Russia was more than 13 years (Human Mortality Database 2016).

National trends in life expectancy are important and have been extensively studied in the region (Leon 2011; Meslé 2004; Meslé et al. 2000; Rychtarikova 2004; Shkolnikov et al. 2001, 2006). Nonetheless, as an indicator of average survivorship, life expectancy conceals considerable heterogeneity in individual mortality trajectories (Edwards and Tuljapurkar 2005; Wilmoth and Horiuchi 1999). This age-at-death variation, hereafter referred to as *life disparity* or *lifespan variation*, is an important dimension of inequality because it summarizes this heterogeneity at the population level and uncertainty in the timing of death at the individual level. Until now, trends in lifespan variation have mostly been studied in the context of mortality decline at all ages (Edwards and Tuljapurkar 2005; Smits and Monden 2009; Vaupel et al. 2011). Alongside increases in life expectancy, ages at death have become more predictable (i.e., lifespan variation has decreased) because mortality decline over young ages has outpaced mortality decline at older ages, compressing most deaths into a narrower age window (Vaupel et al. 2011).

This need not be the case. At the subpopulation level, numerous instances have been documented of lifespan variation increase occurring alongside increases in life expectancy (Brønnum-Hansen 2017; Sasson 2016; Seaman et al. 2016; van Raalte et al. 2014), mainly because of stalls in working-age mortality decline occurring alongside continued old-age mortality decline. To date, no comprehensive studies have explored lifespan variation under periods of life expectancy decline.

We complement the literature by focusing on the CEE case, which shows atypical periods of mortality upheaval and substantial life expectancy changes. This region is particularly interesting because its age pattern of mortality change was very different from that observed in Western countries (Meslé 2004). Given that the largest deviations in age-specific mortality occurred over working ages (Rehm et al. 2007), it is *a priori* unclear what the net effect would be on variability. We analyzed how lifespan variation has changed since the 1960s for 12 countries from this region and determined the ages and causes of death that contributed the most to the observed change in lifespan variation, with a particular focus on the impact of alcohol-attributable mortality.

## Data and Methods

## Dispersion Measure and Demographic Methods

For each population, we investigated life expectancy and lifespan variation since birth. We did not analyze variability at death conditioned on survival to a childhood age, as previous studies have done (e.g., Edwards and Tuljapurkar 2005; Smits and Monden 2009) because of the arbitrariness of choosing a starting age and because infancy and childhood are major contributors to lifespan inequalities that we did not want to overlook. We focused on five periods determined by trends in the coefficient of variation of male life expectancy: (1) *stagnation*, from 1960 to 1980; (2) *improvements*, from 1980 to 1988; (3) *deterioration*, from 1988 to 1994; (4) *divergence*, between 1994 and 2000; and (5) *convergence* thereafter. Periods were initially determined using a divisive hierarchical estimation algorithm for multiple change-points analysis.^1^ The statistical break points were 1960, 1976, 1986, 1993, and 2001. To ease the interpretation of the results, we instead used complete decades or historical events, which were all within three years of the cut points. For example, the period 1960–1979 (complete years) included the two decades with no substantial changes in the coefficient of variation between life expectancies. The next break point (1986) was extended to 1988 to include the entirety of Gorbachev’s anti-alcohol campaign, which was implemented in the period 1985–1988. The following break point (1993) was used exactly because it allows the period 1988–1993 to include the dissolution of the Soviet Union in late 1991 as well as the largest drops in life expectancy in Russia, Latvia, Estonia, and Lithuania and the less marked drops in Ukraine, Belarus, and Bulgaria in 1992–1993. Finally, the year 2001 was changed to 2000 to coincide with start with the twenty-first century.

Several dispersion measures have been proposed to analyze lifespan variability (van Raalte and Caswell 2013; Wilmoth and Horiuchi 1999). In this study, we used life disparity (*e*^†^) as a dispersion indicator (Vaupel and Canudas-Romo 2003). Life disparity is defined as the average remaining life expectancy when death occurs, or life years lost due to death. For example, when death is highly variable, some people will die well before their expected age at death, contributing many lost years to life disparity. When survival is highly concentrated around older ages, the difference between the age at death and the expected remaining years decreases, and life disparity decreases. It can be expressed as

1 $$ {e}^{\dagger }={\int}_0^{\omega }d(a)e(a) da, $$

where *d*(*a*), ω, and *e*(*a*) are the deaths distribution, the open-aged interval (110+ in our case), and remaining life expectancy, respectively.^2^ We selected this measure because of its easy public health interpretation as the average life expectancy losses attributable to death (Shkolnikov et al. 2011) and because of its decomposable and additive properties (Zhang and Vaupel 2009). The *e*^†^ measure has the additive property that after it has been decomposed by age between two periods, the sum of every age-specific contribution to the difference is the total change in *e*^†^ between these two periods. These properties allow us to quantify the impact of mortality at different ages and from different causes and to separate ages that decrease lifespan variability from those that increase it by using demographic methods (Shkolnikov et al. 2011; Zhang and Vaupel 2009). An important attribute of *e*^†^ is the so-called threshold age at which mortality improvements have zero effect on lifespan variation (Zhang and Vaupel 2009). Progress in saving lives below this age reduces variation (also called premature deaths), whereas progress above this age increases variation in lifespans (Vaupel et al. 2011).

The decomposition method used here is based on the line integral model (Horiuchi et al. 2008). Suppose that *f* (e.g., *e*^†^) is a differentiable function of *n* covariates (e.g., each age-cause specific mortality rate) denoted by the vector ***A*** = [*x*_1_, *x*_2_, . . . , *x*_*n*_]^*T*^. Assume that *f* and ***A*** depend on the underlying dimension *t*, which is time in this case, and that we have observations available in two time points, *t*_1_ and *t*_2_. Assuming that ***A*** is a differentiable function of *t* between *t*_1_ and *t*_2_, the difference in *f* between *t*_1_ and *t*_2_ can be expressed as follows:

2 $$ {f}_2-{f}_1=\sum \limits_{i=1}^n{\int}_{x_i\left({t}_1\right)}^{x_i\left({t}_2\right)}\frac{\partial f}{\partial {x}_i}d{x}_i=\sum \limits_{i=1}^n{c}_i, $$

where *c*_*i*_ is the total change in *f* (e.g., *e*^†^) produced by changes in the *i*th covariate, *x*_*i*_. The *c*_*i*_s in Eq. (2) were computed by numerical integration following the algorithm suggested by Horiuchi et al. (2008) and implemented by Riffe (2018). This method has the advantage of assuming that covariates change gradually along the time dimension.

We decomposed changes in life expectancy and lifespan variation by single age, period, and cause of death. For the age-cause decomposition, we used the five-year age group mortality rates from the Human Cause-of-Death Database (2016). All the calculations were performed using R (R Core Team 2000) and are fully reproducible with the available code^3^ and additional information.

The close relationship with other lifespan variation indices, such as Keyfitz’s life table entropy (Vaupel and Canudas-Romo 2003), and the high correlation between them suggest that conclusions would likely be the same regardless of the measure chosen (van Raalte and Caswell 2013; Vaupel et al. 2011; Wilmoth and Horiuchi 1999).

### Data

We used all-cause death counts, population exposures, and period life tables from the Human Mortality Database (2016) for 12 countries from 1960 to the most recent year available in the data set. The countries included in the study were from what will subsequently be referred to as (1) Central Europe (Bulgaria, Czech Republic, Hungary, Poland, Slovakia, and Slovenia), (2) the Baltic countries (BC: Estonia, Latvia, and Lithuania), and (3) other FSU countries (Belarus, Russia, and Ukraine). Data for Slovenia were available only from 1983. The data are by single age, year, sex, and country.

Cause-of-death data came from the newly developed Human Cause-of-Death Database (2016), which provides coherent cause-specific mortality data time series from 1994 to 2010 for eight of the countries in the study (Belarus, Czech Republic, Poland, Russia, Ukraine, Estonia, Latvia, and Lithuania). A universal and standardized methodology was undertaken to redistribute deaths between 104 disease categories in five-year age groups for inclusion in the database. We used these data to obtain the cause-specific proportion by five-year age groups. This procedure effectively eliminated ruptures surrounding revisions of the International Classifications of Disease (ICD) and substantially reduced cross-country comparability problems owing to different coding practices, particularly from the use of ill-defined and unknown causes. We truncated the cause-of-death analysis at age 85 because of classification quality and presence of comorbidities, and we focused on the period after 1994 because comparable information is available for the eight countries (Human Cause-of-Death Database 2016). Furthermore, we focus on this period because it coincides with the beginning of the divergence in Eastern European mortality trends, particularly between the FSU and Central European countries (Meslé 2004).

#### Cause-of-Death Classification

We grouped causes of death into the following broad categories, with a harmonized time series from 1994 to 2010: deaths wholly attributable to alcohol, circulatory disease, transport accidents, other external causes, infectious and respiratory diseases, cancers, and the remaining causes. For details on the ICD-10 codes for each cause, see Table 1.

**Table 1:** Classification of causes of death amenable to alcohol consumption

Category	ICD-10 Codes
1. Alcohol-Attributable Conditions	
Mental and behavioral disorders due to use of alcohol, alcoholic liver disease and cirrhosis of the liver, or poisoning by exposure to alcohol	F10, K70 and K74, X45
2. Amenable to Alcohol Consumption	
Cardiovascular diseases (ischemic heart diseases, stroke, rheumatic heart diseases; essential hypertension; hypertensive disease; pulmonary heart diseases; nonrheumatic valve disorders; cardiac arrest; heart failure; other heart diseases; sequelae of cerebrovascular disease; diseases of arteries, arterioles and capillaries, other circulatory diseases) and transport accidents	I20–I25, I60–I67 and G45, I00–I09; I10; I11–I15; I26–I28; I34–I38; I46; I50; I30–I33, I40–I45, I47–I49; I51; I69; I70–I78; I80– I99, and V01–V99
3. Other Conditions Amenable to Alcohol Consumption	
Other external causes (accidental exposure to smoke, fire and flames; accidental poisoning by other substance; suicide and self-inflicted injuries; assault; event of undetermined intent; complication of medical and surgical care, accidental falls, accidental drowning and submersion, other accidental threats to breathing, or other accidents and late effects of accidents)	(X00–X09; X40–X44, X46–X49; X60–X84; X85–Y09, Y35, Y36; Y10–Y34; Y40–Y84, W00–W19, W65–W74, W75–W84, W20–W64, W85–W99, X10–X39, X50–X59, Y85–Y91, Y95–Y98)
4. Residual Causes	
Remaining conditions and mortality above age 85	

Our objective in classifying disease was twofold. First, we aimed to see which broad causes of death were the important drivers in changing life disparity levels over the period. Second, knowing that injurious alcohol consumption has long been identified as a major determinant of premature mortality in Eastern European countries, particularly of the FSU (Grigoriev and Andreev 2015; Leon et al. 1997; McKee and Shkolnikov 2001; McKee et al. 2005; Rehm et al. 2007; Zaridze et al. 2009, 2014), we aimed for a classification that could at least partially shed light on mortality change due to changing alcohol patterns and mortality change owing to improvements in lifestyle and medical care.

Attributing mortality to alcohol is not straightforward. Unlike smoking, heavy alcohol consumption can have both immediate and cumulative impacts on mortality. In any period, certain causes (for instance, traffic accidents or alcohol poisoning) may change immediately in response to changing consumption patterns; others (for instance, liver cirrhosis) mainly reflect past consumption behavior (Menon et al. 2001; Rehm et al. 2003), and still others (such as ischemic heart disease, which is a component of circulatory disease) have been implicated in both immediate binge drinking mortality (Kauhanen et al. 1997) and elevated mortality risks from long-term heavy drinking (Roerecke and Rehm 2014). Thus, using an attribution method based on cause of death is sensible only for countries with relatively stable temporal patterns of alcohol consumption (Kraus et al. 2015; Martikainen et al. 2014), which is certainly not the case in our study.

Instead, we grouped causes by the degree to which they associate with alcohol consumption and abuse and other large categories that have undergone major changes through the epidemiologic transition. Deaths wholly attributable to alcohol refer to those health conditions that, by the ICD definition, identify alcohol consumption as a necessary cause and that previous research has identified as wholly attributable to alcohol consumption (Rehm et al. 2010). We also include liver cirrhosis in this first category because approximately three-quarters of deaths from this cause in the region are thought to be attributable to alcohol (Rehm et al. 2003), and it is common practice to include it as a condition attributable to alcohol consumption (Rehm et al. 2003, 2010). However, circulatory disease and transport accidents are also amenable to alcohol consumption, meaning that although many of these deaths do not relate to alcohol, changes in hazardous alcohol consumption would be expected to increase or decrease the baseline levels. As such, we pay careful attention to when these two causes co-move with large changes in causes wholly attributable to alcohol. Although additional rare causes of death can be linked to alcohol consumption, we do not include them in our study because their absolute contributions to mortality change are likely to be very small in the set of countries that we study (Grigoriev and Andreev 2015).

We present our results on CEE males only. Mortality change was larger and more abrupt among men, which more clearly illustrates the added value of looking to lifespan variation in times of crisis. In most cases, trends were similar for both sexes, but the magnitude of change was less for females. Full results for females are presented in the online appendix.

## Results

### Age-Specific Rates of Mortality Improvement

**Fig. 1:**
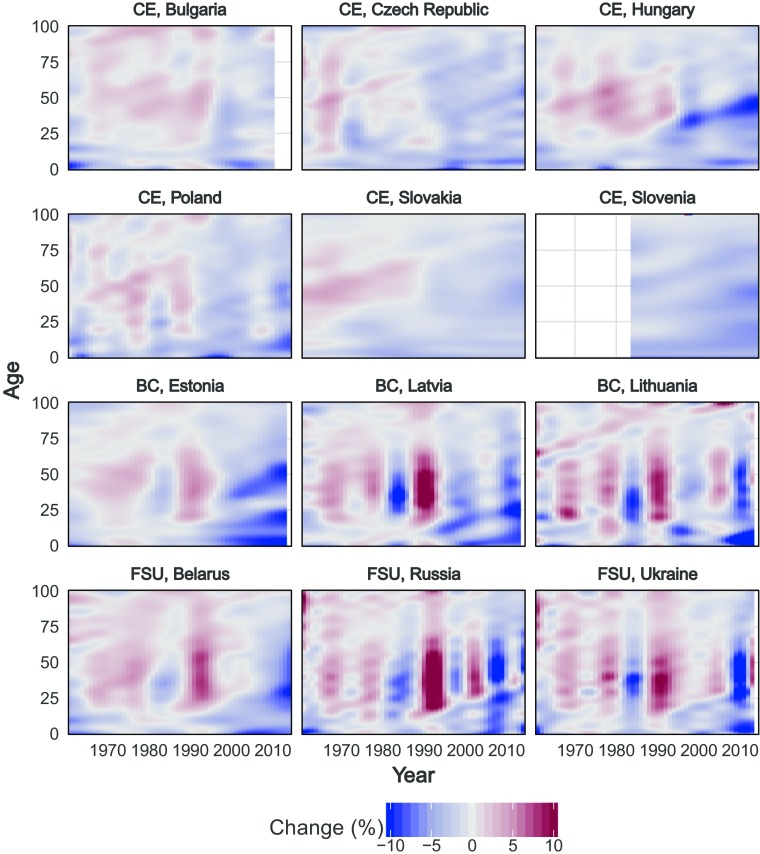
Male mortality surface showing rates of mortality improvements. The regular white areas indicate no data available. *Source:* Own calculations based on Human Mortality Database (2016) data

For a descriptive look at age-specific mortality change over the period, we first examined the average annual rate of mortality improvement (Rau et al. 2013) with smoothed mortality surfaces (Camarda 2012) for males in the 12 CCE countries (see Fig. 1). The respective values are expressed in percentages. Little change or no improvement (–0.5 % to 0.5 %) is depicted in white. Improvement in mortality (i.e., mortality decline) is shown in blue, and mortality increase is shown in red. Darker tones indicate major changes in mortality rates.

Almost every country experienced a near-continuous period of increasing mortality rates, from the mid-1960s to the mid-1980s. Mortality rate increases were mainly concentrated in the ages between 20 and 80 years. After 1985, mortality decreased for a period of approximately five years, most sharply in the BC and the FSU. Opposing this trend, in the early 1990s, the same countries that had made the most progress in reducing mortality experienced intense mortality increases, particularly over working ages. Finally, after the mid- to late 1990s, trends in the region diverged: countries in Central Europe (CE) experienced mortality reduction over nearly every age, as did Latvia and Estonia in the BC. Russia, Ukraine, and Lithuania experienced a second sharp period of mortality increase over working ages in the early 2000s, and age-specific trends in Belarus fell somewhere in between the BC and other FSU countries. Since the mid-2000s, all countries have experienced mortality improvement.

### Trends in Life Expectancy and Lifespan Disparity

**Fig. 2:**
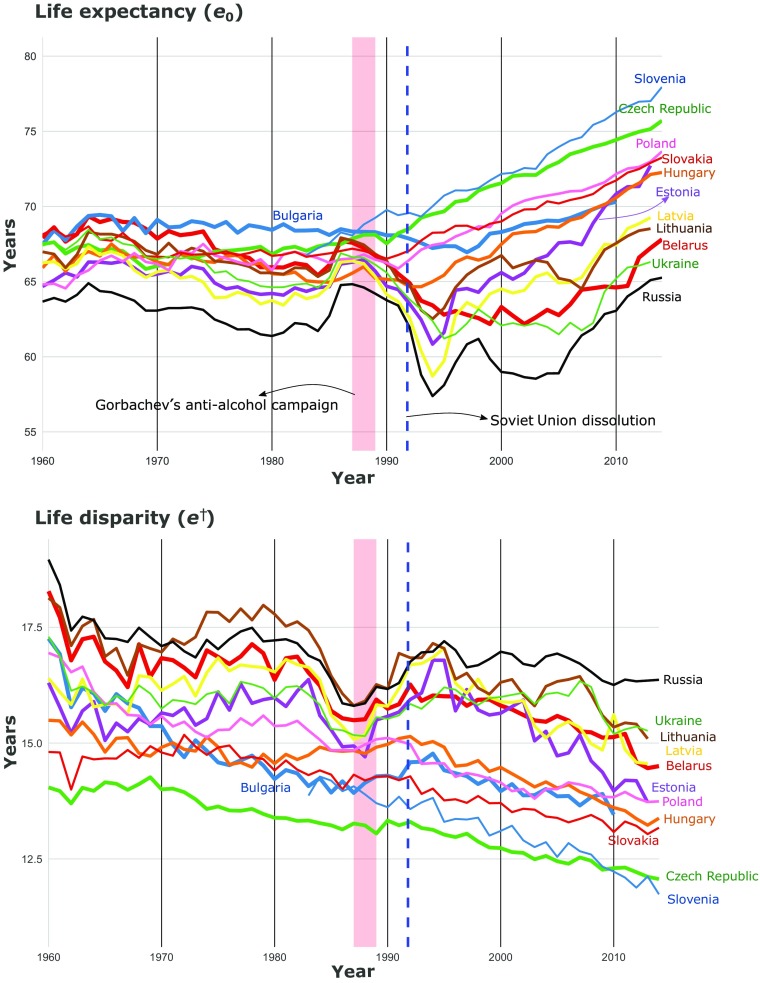
Trends in male life expectancy (*e*_0_) and lifespan disparity (*e*^†^) for 12 Eastern European countries, 1960–2014. *Source:* Own calculations based on Human Mortality Database (2016) data

Figure 2 shows male *e*_0_ and *e*^†^ trends for CEE countries from 1960 to the most recent year available. From 1960 to 1984, *e*_0_ stagnated for most of the countries, and some of them even experienced a slow and steady decline (e.g., Russia, Latvia, Estonia, and Ukraine). This period was followed by a notable increase in *e*_0_ in the mid-1980s, closely corresponding to (although sometimes preceding) Gorbachev’s anti-alcohol campaign, shaded in red. However, after 1987, life expectancy among these countries started to diverge: CE countries experienced a short period of stagnation or decline followed by an upward trend until the end of the study. The BC and other FSU countries experienced a marked decrease in *e*_0_ from 1988 to 1994. Thereafter, *e*_0_ improved everywhere except Lithuania and the other FSU countries. These last countries exhibited a final decrease (Russia and Lithuania) or stagnation (Belarus and Ukraine) in *e*_0_ between 1998 and the mid-2000s, followed by sharp increases in the final period from the mid-2000s to the latest available year. Estonia experienced particularly rapid improvements in *e*_0_ since the mid-1990s, especially among women (online appendix, Fig. A2).

Life disparity showed similar patterns of stagnation between 1960 and 1980 as was seen for *e*_0_. Russia and Lithuania exhibited the highest levels in this period, between 17 and 19 years lost due to death; the Czech Republic presented the lowest level throughout the same years, between 13 and 14 years lost due to death. Importantly, the Czech Republic was not the regional record *e*_0_ holder during these years. Around the mid-1980s, all countries but Hungary experienced compression of mortality (i.e., decreases in *e*^†^). After 1991, Russia and the BC experienced significant increases in *e*^†^, with the peak in 1994–1995. During this peak, the observed *e*_0_ levels differed from historic levels observed when *e*^†^ was equally high. CE experienced continuous reductions in *e*^†^ after 1994, whereas it was less systematic in Latvia and Lithuania. The remaining countries also experienced declines after that year up to 2010–2014 but with greater fluctuation. These declines, however, were not as steep as the *e*_0_ increases in these countries.

### Absolute and Relative Changes in Life Expectancy and Lifespan Disparity

Contrasting the changing levels of *e*_0_ and *e*^†^ from Fig. 2 suggests that in periods of stagnation and mortality upheavals, similar levels of *e*_0_ do not correspond to similar levels in *e*^†^. Therefore, we analyzed the direction and magnitude of change in the two measures.

**Fig. 3:**
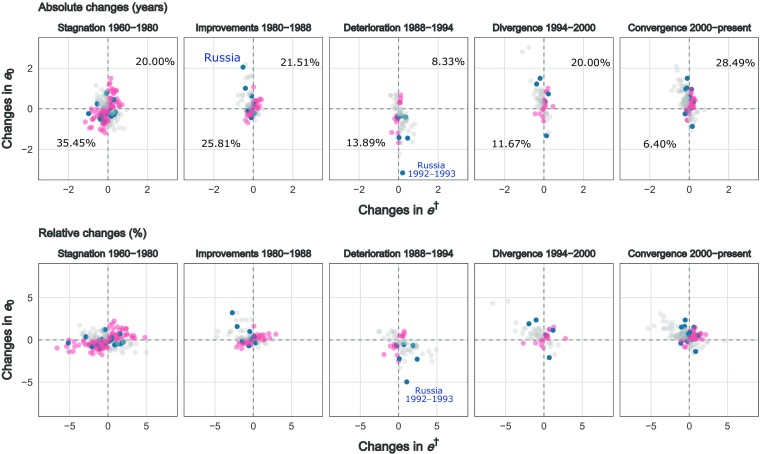
Absolute and relative yearly changes in life expectancy and lifespan disparity, 1960–2010. Data for Slovenia begin in 1983. The black dots are related to changes experienced in Russia. The percentages correspond to the total changes occurred during each period. *Source:* Own calculations based on Human Mortality Database (2016) data

Figure 3 depicts absolute and relative yearly changes (first differences) in *e*_0_ and *e*^†^ for males by period. The periods are grouped by the changes in life expectancy trends depicted in Fig. 2: stagnation^4^ from 1960 to 1980, improvements from 1980 to 1987, deterioration from 1987 to 1994, divergence from 1994 to 2000, and convergence over the period 2000–2010. If a negative relationship existed between *e*_0_ and *e*^†^, changes would concentrate in the top-left and bottom-right quadrants. If points fell in the top-right and bottom-left quadrants, the relationship was positive. We focus on the latter changes and quantify their frequency in three different periods relating to overall mortality trends. Gray dots correspond to a negative association between life expectancy and life disparity (e.g., increases in *e*_0_ with decreases in *e*^†^), and red dots correspond to a positive association (e.g., increases in *e*_0_ with increases in *e*^†^). Because Russia is both the largest country included in the analysis and the country with the most volatile mortality trends, we marked its points in dark blue. Absolute changes (top panel) are easy to interpret because they reflect the changes in life expectancy and life disparity measured in years. However, because the maximum value of *e*_0_ is much higher than the maximum value of *e*^†^, it is not surprising that changes vary more strongly on the *e*_0_ axis than the *e*^†^ axis. Therefore, it is also important to analyze changes in both measures in relative terms (bottom panel), which allows us to quantify the intensity of such changes.

During 1960–1980, almost one-third of the yearly changes in mortality resulted in decreases in both *e*_0_ and *e*^†^ for both males (35.5 %; 95 % CI = [29.1,41.8]) and females (32.7 %; 95 % CI = [26.5,38.9]). These were mostly small changes, corresponding to less than one year of life. Conversely, 20.0 % (95 % CI = [14.7,25.3]) (for males) and 24.6 % (95 % CI = [18.9,30.2]) (for females) of *e*_0_ increases corresponded to *e*^†^ increase. This means that when both quadrants are added, the measures in this period were moving in the same direction more than half the time. A similar pattern was observed in the period 1980–1988. In 1988–1994, when most of the changes corresponded to substantial decreases in *e*_0_, the two indices moved in the same direction approximately one-fifth of the time. Finally, in the period 1994 onward, characterized by mortality convergence, approximately one-third of all points related to movements in the same direction for both measures.

Moreover, even when the two measures moved in the direction expected from a negative correlation, the magnitude of change in life expectancy did not reflect the same magnitude of change in life disparity. For example, Russia lost three years of male life expectancy (approximately 5 %) between 1992 and 1993, but life disparity showed a much smaller increase (less than 2.5 %). Most of the time, however, *e*^†^ experienced larger relative changes than *e*_0_, as evidenced by more movement along the horizontal than the vertical axis in the bottom panel of Fig. 3.

### Age-Specific Decomposition

**Fig. 4:**
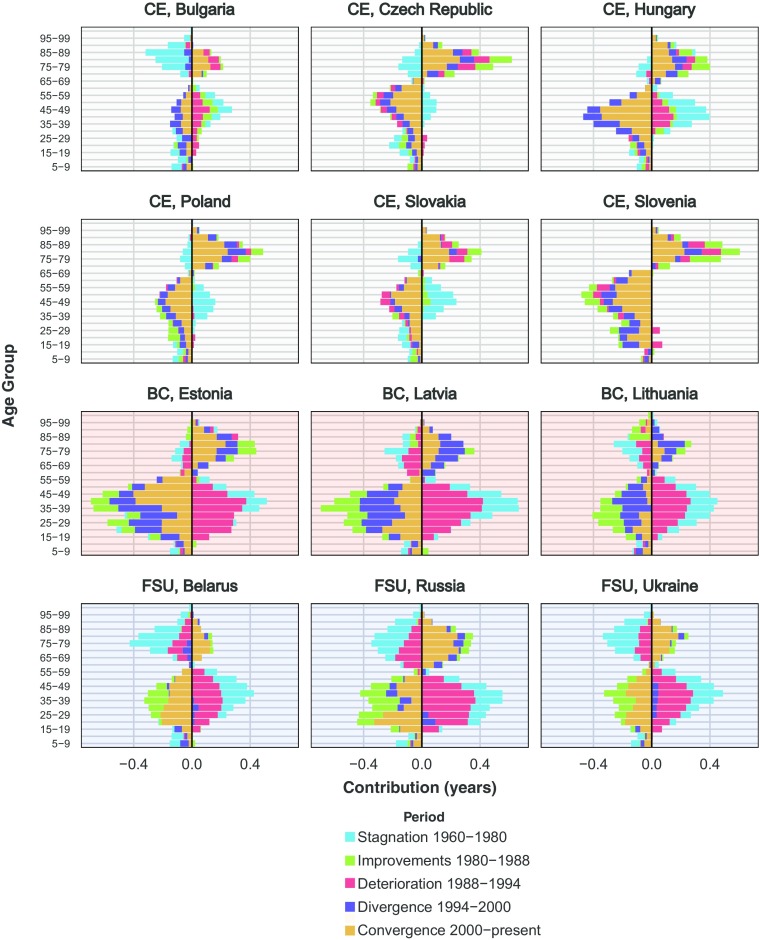
Males’ age-specific contributions to the change in lifespan disparity *e*^†^ by periods. Data for Slovenia begin in 1983. *Source:* Own calculations based on Human Mortality Database (2016) data

**Fig. 5:**
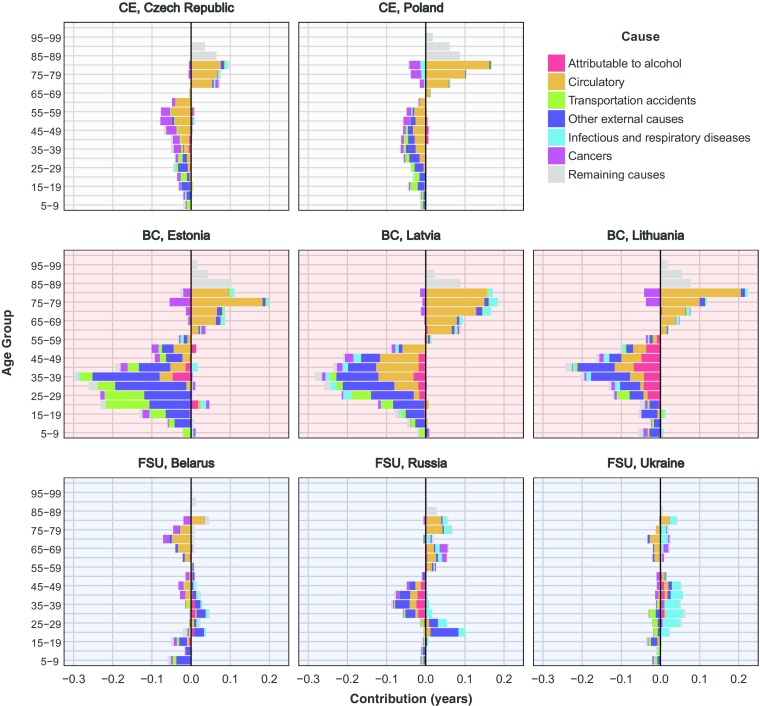
Cause specific contributions to the change in male lifespan disparity *e*^†^, 1994–2000. Data for Poland end in 2009. *Source:* Own calculations based on Human Cause-of-Death Database (2016) data

**Fig. 6:**
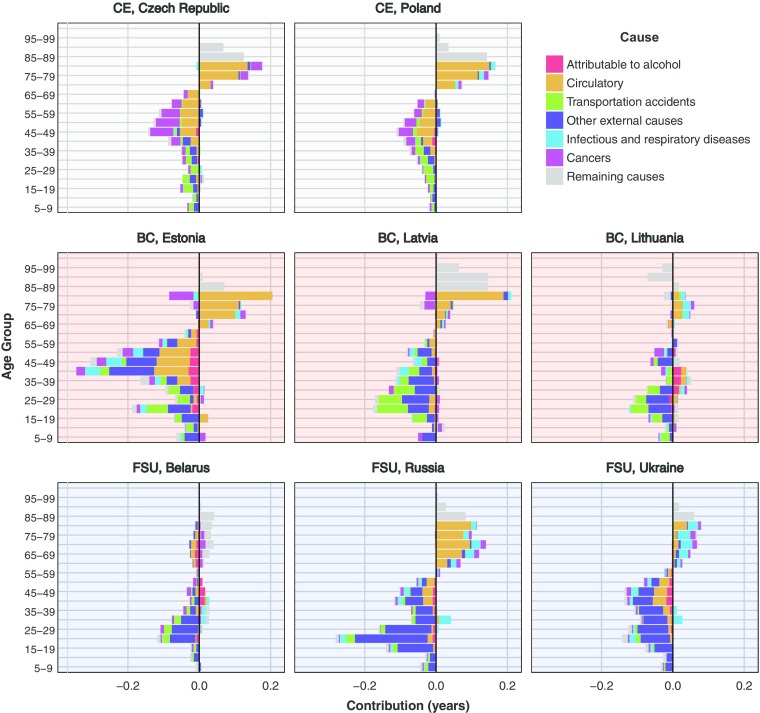
Cause-specific contributions to the change in male lifespan disparity *e*^†^, 2000–2010. Data for Poland end in 2009. *Source:* Own calculations based on Human Cause-of-Death Database (2016) data

In Figs. 4, 5, and 6, countries are ordered alphabetically within each region (CE, BC, and FSU) and differentiated by the background color: light gray for CE, light red for BC, and light blue for other FSU countries. Figure 4 shows age-specific contributions to the change in *e*^†^ for ages 5 and above,^5^ respectively, by period (results for ages 0–4 are depicted in Fig. A7 in the online appendix).^6^ The periods are the same as in the previous figure: stagnation (blue), improvements (green), deterioration (red), divergence (purple), and convergence (orange). The threshold age occurred around the age groups where changes in lifespan variation were usually the lowest by period (e.g., Russia ages 55–59, Slovakia ages 65–69, and Slovenia ages 70–74). Bars on the left (decreases in variation) came about from mortality decreases at young ages or increases at old ages, separated by the threshold age. Conversely, bars on the right (increases in variation) were produced by mortality increases at young ages or mortality decreases at old ages. Colors lining up on one side would suggest that mortality changed in different directions for younger compared with older ages.

Over the long period of *e*_0_ stagnation (blue), changes in *e*^†^ were driven by mortality increases at all ages above approximately age 20, which expanded variability in age at death at young-adult ages and compressed variation at older ages in all countries. It is worth noting that these changes mostly offset each other: the old-age compression was comparable with the net expansion of mortality experienced by children and younger adults. In fact, in Bulgaria and Belarus, the compression caused by mortality increases over older ages was greater than the expansion made by mortality increases among younger ages.^7^ A similar pattern was observed during the period of *e*_0_ deterioration among BC and other FSU countries following the collapse of the FSU (red). Lifespan variability mostly increased, which was explained by expansion of mortality at young and middle ages, alongside smaller compression at older ages during this period. By contrast, CE countries experienced little change in mortality during this period.

Opposing these trends, over the period of improvements during the 1980s (green), the BC and other FSU countries followed a Western pattern, with *e*^†^ decreases mostly caused by mortality decline at younger ages outpacing mortality decline over older ages and leading to overall compression in mortality. Mortality change was smaller, and the age patterns of change were more variable in CE during this period. From 1994 onward (purple and orange), all countries experienced *e*^†^ compression at younger ages and expansion at older ages overall. However, in the early years (1994–2000), mortality increases at younger ages led to increases in *e*^†^ in FSU before reversing itself in recent years. Importantly, during this post-1994 period, mortality changes at relatively young ages (20–50) had the largest impact on *e*^†^ changes.

### The Contribution of Different Causes of Death to Changes in Lifespan Variability

Table 2 shows the net contribution of different broad causes of death to changes in life disparity for the most recent periods of divergence (1994–2000) and convergence (2000–2010). From 1994 to 2010, all the countries included in our study experienced decreasing *e*^†^. Life disparity was reduced by nearly a year in CE, equally spread between both periods, and owing primarily to mortality from transport accidents and cancers. In BC, *e*^†^ declined by between 1.2 years (in Latvia) and 2.8 years (in Estonia). Declines were strong over both periods, driven by transport accidents, other external causes, and mortality wholly attributable to alcohol (Lithuania, 1994–2000 only). Finally, the other FSU countries showed little change in *e*^†^ over the earlier period and strong declines in the second period. Like with the other country groupings, changes in mortality from other external causes seemed to be driving net changes in *e*^†^. Belarus was the only country to experience increases in *e*^†^ from causes of death that were wholly attributable to alcohol.

**Table 2:** Cause-specific contributions to the change in *e*^†^ for males, 1994–2000 and 2000–2010

Period	Group	Country	Mortality Attributable to:							
			Alcohol	Circulatory	Other External Causes	Transport Accidents	Infectious and Respiratory	Cancers	Rest	Total
1994–2000	CE	Czech Republic	0.01	–0.04	–0.09	–0.06	0.01	–0.16	–0.07	–0.40
		Poland	0.01	0.15	–0.18	–0.08	–0.07	–0.13	–0.11	–0.41
	BC	Estonia	–0.04	0.30	–0.78	–0.41	–0.01	–0.14	–0.03	–1.11
		Latvia	–0.11	0.15	–0.64	–0.14	–0.12	–0.09	–0.01	–0.96
		Lithuania	–0.28	0.21	–0.39	–0.04	–0.09	–0.15	–0.09	–0.83
	FSU	Belarus	0.03	–0.13	0.01	–0.03	0.01	–0.10	0.01	–0.20
		Russia	–0.07	0.10	–0.02	–0.03	0.09	0.00	–0.08	–0.01
		Ukraine	0.04	–0.03	–0.04	–0.07	0.21	–0.02	–0.05	0.04
2000–2010	CE	Czech Republic	–0.01	–0.00	–0.11	–0.14	0.00	–0.23	0.04	–0.45
		Poland	–0.01	0.11	–0.06	–0.16	0.01	–0.16	–0.05	–0.32
	BC	Estonia	–0.17	0.03	–0.60	–0.23	–0.14	–0.15	–0.41	–1.67
		Latvia	–0.01	0.17	–0.47	–0.34	–0.04	–0.02	0.43	–0.28
		Lithuania	0.05	0.12	–0.35	–0.21	0.01	–0.03	–0.53	–0.94
	FSU	Belarus	0.02	–0.07	–0.33	–0.10	0.01	–0.02	–0.17	–0.66
		Russia	–0.06	0.28	–0.70	–0.10	–0.03	0.01	–0.12	–0.72
		Ukraine	–0.08	–0.09	–0.53	–0.04	0.05	–0.01	–0.10	–0.80

Figure 5 shows these cause-of-death contributions by age for 1994–2000.^8^ The sum of the age-specific contributions by causes of death results in the values shown in Table 2. In CE countries, mortality decline was driven predominately by circulatory disease, but because these declines were spread before and after the threshold age, the net impact on *e*^†^ was small. Causes that were completely attributable to alcohol showed no change over the period, and reductions in external-cause mortality were comparatively minor, which suggests that reductions in circulatory disease mortality were not related to changing alcohol consumption in the region. In BC, the reduction in circulatory disease mortality was strong overall but particularly over older ages, explaining why its net contribution over all ages was to increase *e*^†^. Declines in mortality from external causes, including traffic accidents, below age 50 were the largest contributors to *e*^†^ decline. Alcohol-attributable mortality over these ages also declined, especially in Lithuania. Taken together, these findings suggest that reductions in hazardous alcohol consumption played some role in reducing *e*^†^ over the period in BC. Finally, in the other FSU countries, mortality declines over all ages were weaker, whereas trends in major causes of death were inconsistent over age.

From 2000–2010, all countries experienced improvements in survival and decreases in *e*^†^, although from a different mixture of causes (Fig. 6). In CE, reductions in early adult cancers and circulatory disease predominated; reductions in mortality from external causes, including traffic accidents, were of secondary importance. The BC were heterogeneous over this period: Estonia experienced sharp reductions in mortality from circulatory diseases at all ages and external causes below age 50. Lithuania experienced virtually no change in circulatory disease mortality, some decrease in external-cause mortality below age 30, and increases in mortality attributable to alcohol over ages 30–44. Latvia fell somewhere between the two countries. Life disparity also declined in the other FSU countries, mainly because of declines in external-cause mortality. However the *e*^†^ declines were noticeably lower in Ukraine, while circulatory diseases there actually increased at older ages; by contrast, Russia and Ukraine experienced sharp and moderate declines in circulatory disease mortality at these older ages.

## Limitations

The limitations of our study should be mentioned. First, different measures of inequality differ in formal properties and in the degree of sensitivity to age-specific mortality change (van Raalte and Caswell 2013). Other authors have chosen measures of relative inequality, such as the Gini coefficient, Keyfitz’s entropy, or the Theil index of inequality (Colchero et al. 2016; Moser et al. 2005; Shkolnikov et al. 2003; Smits and Monden 2009). As a robustness check, we performed a sensitivity analysis replicating all the results shown in this study with the Gini coefficient, following Shkolnikov et al. (2003) (see the online appendix). We did not find major differences with the results discussed in this article.

We chose not to decisively partition mortality into alcohol- and non-alcohol-related mortality because of the difficulties in determining the proportion of deaths from circulatory disease and external causes that are related to alcohol. Instead, we took a more cautious approach that aimed to attribute the changes in mortality trends at least partially to alcohol consumption without over- or underinterpreting its absolute impact on mortality, based on the co-movements of these causes with known causes of death that are wholly attributable to alcohol. An alternative would have been to derive alcohol-attributable mortality from follow-up longitudinal studies that report consumption patterns. Even if such surveys were available for some countries included in the study, self-reported alcohol consumption data are often biased and underestimate actual consumption because individuals forget drinking occasions, underestimate drink size, and cannot remember the quantity of drinks in every drinking session (Bellis et al. 2009; Livingston and Callinan 2015). A third commonly used approach is to link mortality with changes in alcohol sales (Razvodovsky 2010). A limitation to this approach is that the total alcohol consumption might not matter as much as the alcohol consumption behavior. Indeed, low levels of alcohol consumed at a regular basis may even be protective against mortality (Bell et al. 2017; Klatsky et al. 1974; Rehm et al. 2010; Roerecke and Rehm 2014). Moreover, alcohol sales do not include homemade alcohol, which is substantial in the region (McKee et al. 2005; Popova et al. 2007) and can be distorted by alcohol tourism (Mäkelä and Österberg 2009; Rabinovich et al. 2009).

There could be concerns with the quality of the data used in a comparative temporal setting. First, the CEE countries used a definition of live births that is less strict than the WHO definition, thus artificially depressing infant mortality levels (Aleshina and Redmond 2005; UNICEF 2003). All countries eventually shifted to the WHO definition, although the timing of this shift differed between and within countries, with some regions beginning the shift even before the dissolution of the FSU (Aleshina and Redmond 2005; Anderson and Silver 1986; UNICEF 2003). Given that indices of lifespan variation are comparatively more sensitive to changes in infant mortality than life expectancy (van Raalte and Caswell 2013), we investigated whether our results would be robust to the following assumptions: (1) a doubling of infant mortality prior to 1990, followed by a linear decrease to 10 % higher rates in 2000, and constant inflation of 10 % thereafter;^9^ and (2) mortality conditional on survival to age 5. Although these scenarios created some differences in the direction of trends—particularly over the communist period, when infant mortality decline was substantial—our two main conclusions from this period still held: (1) life expectancy and life disparity moved independently during the years before the fall of the Berlin Wall, and (2) trends in life disparity were especially driven by trends in early-adult mortality. The results of these robustness checks are available in the online appendix.

Second, the Human Mortality Database (2016) data used in this project are the highest-quality and most-comparable data available for the region. However, the data quality differs across countries, age groups, and periods, and is well documented in the database. The main data quality concerns that have been flagged in the region include (1) age heaping and likely age exaggeration in many FSU countries and Bulgaria in the 1960s (Grigoriev 2017; Jasilionis 2017b; Jdanov and Shkolnikov 2017; Philipov and Jasilionis 2017; Pyrozhkov et al. 2017); (2) lower-quality data above age 80 in Belarus in the 1970s (Grigoriev 2017) and Russia after the mid-1990s (Jdanov and Shkolnikov 2017); and (3) consistency problems in population estimates in Lithuania for the 1960s and 1970s (Jasilionis and Stankuniene 2017), Estonia during the 1990s (Jasilionis 2017a), and Slovenia (Jasilionis 2017c). Age heaping is less of a problem for life table summary measures, but age exaggeration is difficult to correct for and could have led to artificially worsening mortality at older ages as data quality improved. Although a degree of caution should be applied in interpreting mortality differences and trends for these periods, age groups, and countries, even if we were to exclude all instances of these flagged problems, the broader patterns of mortality development documented here still hold.

Third, in the Soviet era, ill-defined cardiovascular diseases were often classified as a*therosclerotic cardiosclerosis*, which is a subset of ischemic heart diseases (Jasilionis et al. 2011; Shkolnikov et al. 2012). Different countries abandoned this practice at different rates, which had the effect of misclassifying deaths between the ischemic heart disease, stroke, and “other circulatory disease” categories. Although some degree of misclassification within circulatory disease is corrected for by the Human Cause-of-Death Database (2016) team (Pechholdová et al. 2017), we thought it was safer for comparative purposes to combine all circulatory disease categories.

Finally, there could be concern about data quality relating to high emigration throughout the post-Soviet period. However, robustness checks conducted for Poland and Czech Republic (Fihel and Pechholdová 2017) and the BC (Jasilionis et al. 2011) showed that underestimated emigration resulted in an overestimation of life expectancy of up to four months in Poland during the intense outflows following accession to European Union, but in other countries and periods, it was usually equivalent to less than one month.

## Discussion

We analyzed and compared a long time series of life disparity for 12 countries from CEE. Decomposing these trends by age and cause of death shed light on the determinants of lifespan variation across time and countries. Over the study period, the acute mortality crises of the 1990s caused greater year-to-year fluctuation in lifespan variation than in life expectancy. Life expectancy and life disparity moved independently of each other, particularly during periods of life expectancy stagnation caused by uneven age-specific mortality change. Changes in life disparity were largely caused by changes in midlife mortality, with different net effects depending on the country and period.

### Changes in Life Expectancy (*e*_0_) and Life Disparity (*e*^†^)

Previous studies have found a strong negative correlation between life expectancy and life disparity when measured over all ages (Colchero et al. 2016; Vaupel et al. 2011; Wilmoth and Horiuchi 1999). These studies were carried out over long periods of 100 years or more and mostly included Western countries with near-monotonic increases in life expectancy. Importantly, two major phenomena were observed from the mid-nineteenth century to the present: (1) a drastic reduction of infectious disease mortality, and (2) a subsequent major decline in cardiovascular disease mortality. These epidemiological changes can equally be considered as a redistribution of deaths from young to middle ages and later from middle to older-adult ages (Robine 2001). In both cases, contemporaneous mortality decline over younger ages (ages that compress mortality into a smaller age interval) outpaced decline over older ages (ages at which mortality decline leads to deaths occurring over a larger age interval), which caused life disparity to decrease in lockstep with life expectancy increase.

CEE countries ran counter to this narrative. Although they too experienced the sharp declines in infectious disease mortality up to the mid-twentieth century, mortality at midlife stalled or even increased for most of the last half of the twentieth century (McKee and Shkolnikov 2001), with no appreciable declines in cardiovascular mortality until very recently (Caselli et al. 2002; Grigoriev et al. 2014; Meslé 2004; Timonin et al. 2017). As our results made clear, mortality change at different ages was far from even, such that changes in lifespan variation did not correspond in intensity or in the desirable direction with changes in life expectancy (i.e., an increase in life expectancy with a decrease in lifespan variation). For example, it was apparent that between-country differences in lifespan variation have and continue to be larger (in relative terms) than between-country differences in life expectancy (coefficients of variation for *e*_0_ and *e*^†^ in 2014 are 0.06 and 0.11, respectively).

From a public health perspective, these results are important because they disclose inequalities underlying population health that could not be identified by looking at life expectancy alone. As noted earlier, the full distribution of deaths is characterized not only by the mean (life expectancy) but also by the dispersion in ages at death (Edwards and Tuljapurkar 2005). Periods of increasing lifespan variability underscore both a rise in within-group heterogeneity at the population level and increasing uncertainty about the timing of death at the individual level. Similar episodes have been found previously for some countries, and they are seen as outliers that are not following the classic Western trend (Wilmoth and Horiuchi 1999). For instance, stagnating or increasing lifespan variation has been seen alongside life expectancy increase among lower socioeconomic groups or regions in Europe, driven by mortality stagnation among young adults (Brønnum-Hansen 2017; Seaman et al. 2016; van Raalte et al. 2014). In the United States, lower-educated groups have experienced both life expectancy decreases and increases in lifespan variation (Sasson 2016). More recently, much attention has been paid to poor trends in midlife mortality among white Americans, particularly females (Case and Deaton 2015; Montez and Zajacova 2013). As Gillespie et al. (2014) noted, the challenge of reducing young-adult mortality could anticipate a new pattern characterized by increases in lifespan inequality. Our results offer further proof of the independence of the two measures during long periods with atypical mortality schedules and illustrate the need to monitor lifespan variation for a complete picture of population health.

At the same time, our results revealed a paradox of sorts. On the one hand, between-country differences in lifespan variation were more stable than between-country differences in life expectancy. On the other hand, changes in lifespan variation were more sensitive to year-to-year mortality fluctuations than life expectancy, particularly when viewed on a relative scale. Measures of dispersion are more sensitive to mortality change in early midlife than life expectancy (van Raalte and Caswell 2013). Mortality between ages 25 and 50 experienced larger changes in response to crises than older-adult mortality over the period (as shown clearly in Fig. 1), which explains why life disparity showed greater year-to-year fluctuation than life expectancy. Meanwhile, mortality differences over older working ages and among the early retired have a larger impact on life expectancy than life disparity: these ages are found on either side of the threshold age, with mortality declines (or increases) often offsetting each other, so that the net impact is no change in lifespan variation. As a result, the combination of mortality changes over younger ages with growing mortality differences at older-adult ages can lead to widening between-country inequalities in life expectancy alongside stable differences in life disparity.

### Cause-of-Death Contributions to Changes in *e*^†^ After 1994

The impact of alcohol on mortality has been extensively studied in Russia, which experienced the largest mortality swings in the region (Leon et al. 1997; Rehm et al. 2007; Shkolnikov et al. 2013, 2001). Alcohol-related mortality is also known to have played an important role in mortality trends since the 1980s in BC and other countries of the FSU (Jasilionis et al. 2011; Rehm et al. 2007), although its specific impact on lifespan variation has not been thoroughly investigated. To date, only Shkolnikov et al.’s (2003) study on Russia between 1979 and 1989 has analyzed the ages and causes of death contributing to changing lifespan variation in the region. They found that mortality compression resulting from a reduction of death rates at early-adult ages during this period was attributed to a decrease in alcohol-related mortality as a consequence of Gorbachev’s anti-alcohol campaign. We extended this cause-of-death analysis to include more countries (Belarus, Czech Republic, Estonia, Latvia, Lithuania, Poland, Russia, and Ukraine) and focused on the 1994–2010 post-Soviet years.

Fluctuating alcohol-related mortality was an important component of the moving life disparity trends in the countries of the former Soviet Union, although it occurred to different degrees in each region and manifested itself in different causes. Over young ages, we found evidence of a large role for the reduction in mortality from external causes, including traffic accidents, in BC throughout the period and in Russia, Belarus, and Ukraine from 2000 onward. That these causes often co-moved with mortality directly attributable to alcohol over these ages is suggestive that healthier patterns of alcohol consumption were contributing to these reductions in life disparity. At older ages, between-country differences in mortality reduction seemed to be driven by the extent of mortality reduction from circulatory diseases. Alcohol consumption was not the only factor that explained mortality trajectories in the region, nor was it the sole explanation for the difference between this region and Western European countries in terms of life expectancy and lifespan variation levels. Other factors, such as environmental pollution, medical care, smoking behaviors, and diet, have been important determinants of health outcomes in this region since at least 1970 (Bobak and Marmot 1996). Indeed, the strong declines in circulatory disease mortality in BC (Jasilionis et al. 2011) and more recently Russia (Grigoriev et al. 2014) have been seen as hopeful signs that these countries are finally on a path toward the lower levels of cardiovascular mortality that have been achieved in the West.

In contrast to BC and other FSU countries, the smoother trends in life disparity found in CE were driven by sustained declines in circulatory disease and cancers, with external causes playing a much smaller role and no change in mortality directly attributable to alcohol. This finding is consistent with the suggestion by other researchers that the steady post-1990 improvements in mortality in the region were attributable to a combination of improvements in medicine, a reorganization of the health care system, and general shifts toward healthier behavior, including improving diets and reductions in smoking (Cífková et al. 2010; Cooper et al. 1984; Fihel and Pechholdová 2017; Nolte et al. 2000a, b; Pajak and Kozela 2011; Rychtarikova 2004; Zatonski et al. 1998). We additionally identified a recent stagnation (since 2010) in lifespan variation in Russia. As our decomposition results after 2010 suggest, this stagnation could be a result of a slowdown in mortality improvements below age 60 offset by larger progress above this age. Timonin et al. (2017) suggested that the stagnation could be a result of uneven progress in reducing cardiovascular mortality at the subregional level in Russia that is offset by convergence in under-60 mortality.

Preventing external-cause mortality at young ages has been previously highlighted as an immediate way to reduce lifespan variability and differences in life disparity between populations. Firebaugh et al. (2014) argued that allocating resources to reducing homicides in the American black population was more likely to narrow racial disparities in lifespan variation than tackling more common causes of death. In this sense, most reductions in life disparity in the region were caused by improvements in mortality at young ages after 1994, particularly in mortality from external causes. The decline in these causes of death also increased life expectancy (Trias-Llimós et al. 2018) and contributed to convergence between countries in the region in lifespan variation, as we showed. Similarly to some developed countries—Canada, France, Germany, Japan, and others (Seligman et al. 2016)—reduction in mortality from cancers and cardiovascular diseases helped increase life expectancy in CEE but did not account for most life disparity reductions.

Mortality associated with the most hazardous forms of alcohol consumption, such as mortality from alcoholic liver disease or poisoning by exposure to alcohol, did not play a central role in lifespan variation levels or trends—perhaps in part because these are small causes of death to begin with in comparison with much larger causes of death, such as circulatory disease or external causes. Nevertheless, some countries (Lithuania, Russia, and Latvia) did show large mortality improvements in these conditions, which caused compression of mortality at young ages. These differences were previously noted as a partial explanation for different mortality trends in Lithuania and Belarus (Grigoriev et al. 2015).

Lifespan variation, in this case *e*^†^, is a measure of aggregate health inequality that reveals fundamental differences in levels and trends across the countries that we studied. Therefore, analyzing lifespan dispersion together with life expectancy contributes to a deeper understanding of the impact of changing mortality trends on population health. Our results show not only that CEE countries experienced high lifespan variation and consequently greater fluctuation in the predictability of lifespan but also that life expectancy and life disparity were able to move independently, particularly in periods of stagnation in life expectancy. These uncommon findings, opposing those observed in most developed countries, show that expansion (compression) levels do not necessarily mean lower (higher) life expectancy or mortality deterioration (improvements) when the yearly changes over time are taken into account.

## Electronic supplementary material

ESM 1 (PDF 2208 kb)

## Footnotes

1 Using the package *ecp* in R.

2 The discrete formula from life table functions used is ${\sum}_{x=0}^{109}\left[d(x)\left(e(x)+a(x)\times \left[e\left(x+1\right)-e(x)\right]\right)\right]+d\left(110+\right)\times e\left(110+\right).$

3 The code is available online (https://goo.gl/wmpXjJ).

4 Even though some countries experienced a steady decline during this period, we labeled the period *stagnation* because the coefficient of variation of life expectancies during this period was stagnant.

5 For age-specific contributions to life expectancy, see the online appendix.

6 Declines in mortality below age 5 were the strongest age-category contributor to changes in life disparity over the period under study, with most of the decline occurring over the 1960–1980 period. For this reason, they are plotted separately from other ages with a different scale used on the *x*-axis in the online appendix. The decline was near monotonic in most countries, with only minor differences between countries, especially when compared with the much larger between-country differences at other ages.

7 Increasing mortality at older ages might have been an artifact due to improvements in data quality (see the Limitations section).

8 For age-cause-specific contributions to life expectancy in the same periods, see the online appendix.

9 Kingkade and Sawyer (2001) published adjustment factors that were generally much lower than the doubling that we used here. Thus, this adjustment should be seen as a conservative rather than realistic adjustment to test the robustness of our findings. For the 1960–1980 period, inflating the infant mortality by anywhere up to 77 % for males and 95 % for females resulted in yearly changes in life expectancy and life disparity moving in unexpected directions up to 50 % (see https://demographs.shinyapps.io/CEE_App/).
