# Supplementary material for: Lifespan Dispersion in Times of Life Expectancy Fluctuation: The Case of Central and Eastern Europe
Source: Demography. 2018 Nov 12;55(6):2071–96. doi: 10.1007/s13524-018-0729-9 (PMC6290692; doi:10.1007/s13524-018-0729-9)
Supplement: Supplementary file 1 — (PDF 2208 kb) [file 13524_2018_729_MOESM1_ESM.pdf]

Online Appendix

Lifespan dispersion in times of life expectancy fluctuation: the case of Central and Eastern Europe

José Manuel Aburto*[1,2] and Alyson van Raalte†[2]

[1]University of Southern Denmark
[2]Max Planck Institute for Demographic Research

Abstract

Central and Eastern Europe (CEE) have experienced considerable instability in mortality since the 1960s. Long periods of stagnating life expectancy were followed by rapid increases in life expectancy and, in some cases, even more rapid declines, before more recent periods of improvement. These trends have been well documented, but to date, no study has comprehensively explored trends in lifespan variation. We improved such analyses by incorporating life disparity as a health indicator alongside life expectancy, examining trends since the 1960s for 12 countries from the region. Generally, life disparity was high and fluctuated strongly over the period. For nearly 30 of these years, life expectancy and life disparity varied independently of each other, largely because mortality trends ran in opposite directions over different ages. Furthermore, we quantified the impact of large classes of diseases on life disparity trends since 1994 using a newly harmonized cause- of- death time series for eight countries in the region. Mortality patterns in CEE countries were heterogeneous and ran counter to the common patterns observed in most developed countries. They contribute to the discussion about life expectancy-disparity by showing that expansion/compression levels do not necessarily mean lower/higher life expectancy or mortality deterioration/improvements.

*jmaburto@health.sdu.dk
†vanRaalte@demogr.mpg.de

Results shown in the paper reproduced for females

Figure A1: Female mortality surface showing rates of mortality improvements

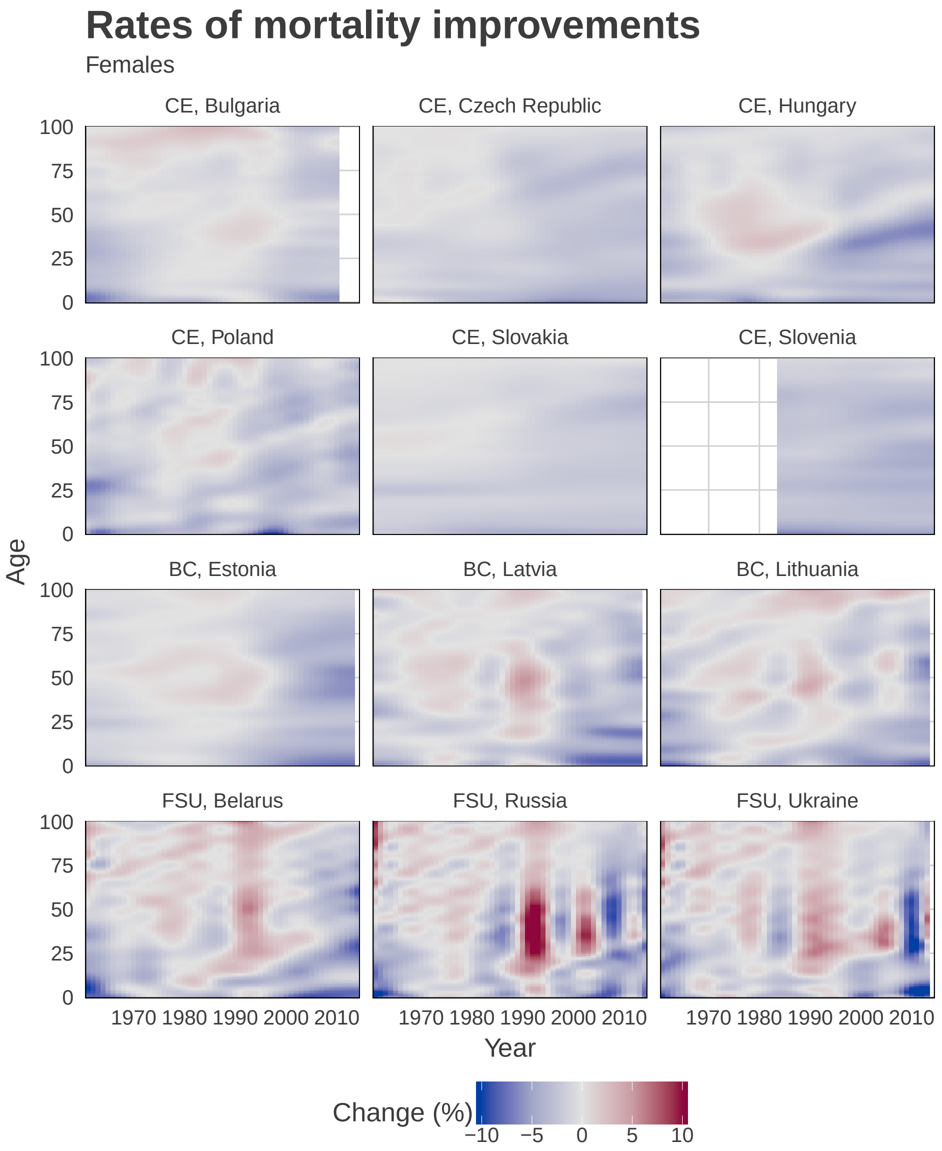

Source: own calculations based on Human Mortality Database (2016) data. Note: White areas indicate no data available

Figure A2: Trends in female life expectancy (e_0) and lifespan disparity ($e^{\dagger}$) for 12 Eastern European countries, 1960-2014

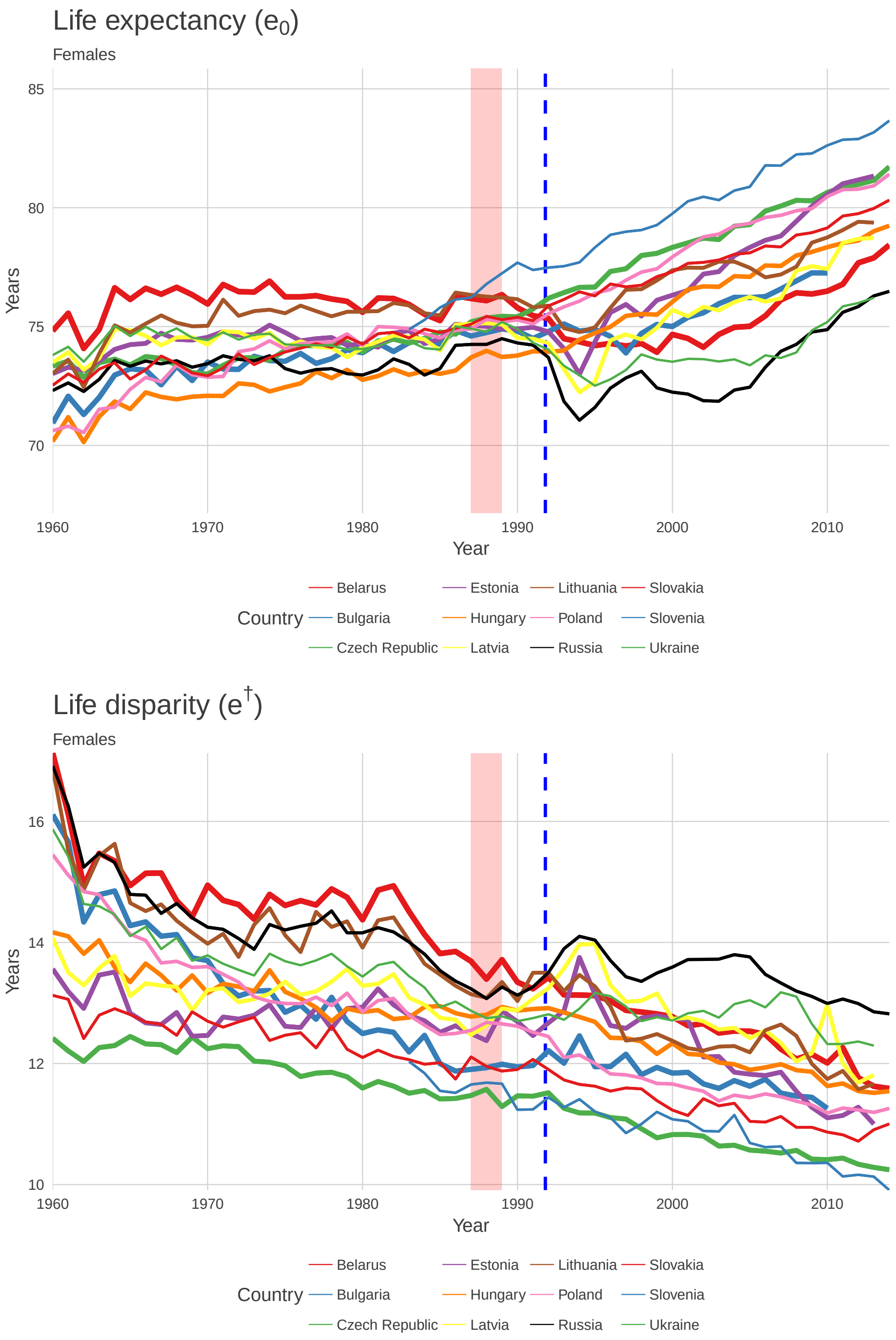

Source: own calculations based on Human Mortality Database (2016) data.

Figure A3: Absolute and relative yearly changes in life expectancy and lifespan disparity for females, 1960-2010

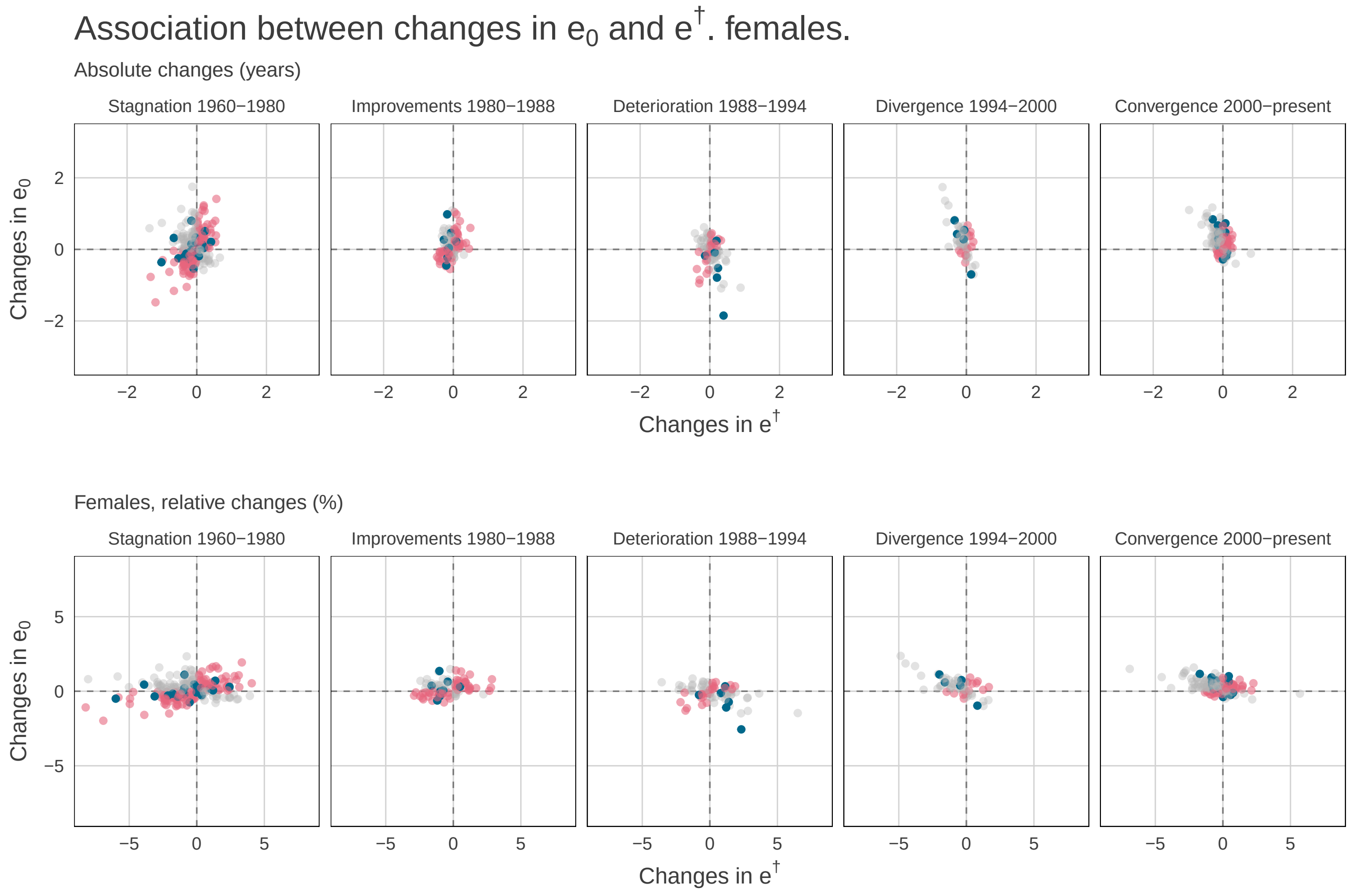

Source: own calculations based on Human Mortality Database (2016) data. Note: data for Slovenia begins in 1983. The dark dots are related to changes experienced in Russia.

Figure A4: Age-specific contributions to the change in lifespan disparity $e^{\dagger}$ by periods, females.

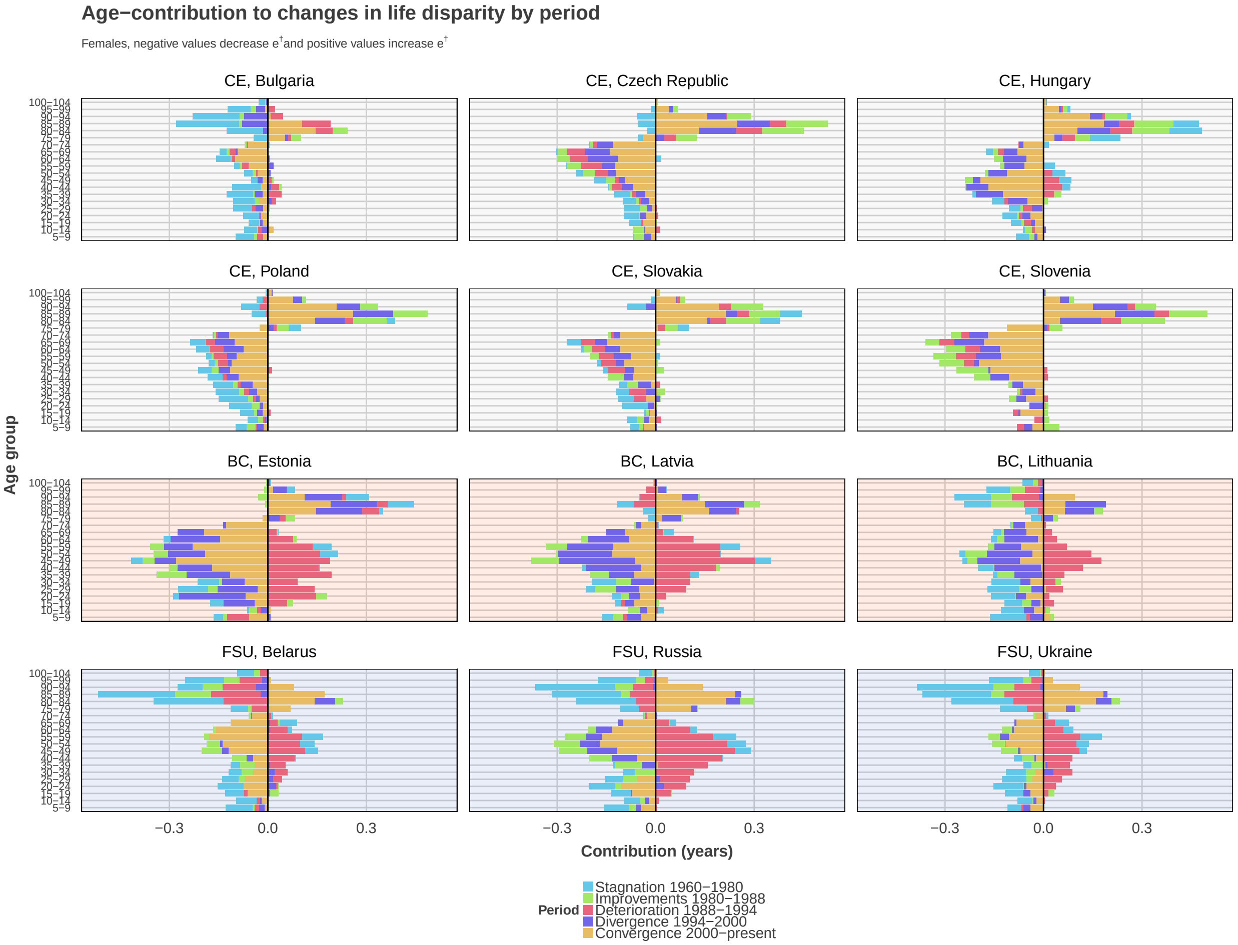

Source: own calculations based on Human Mortality Database (2016) data. Note: data for Slovenia begins in 1983.

Figure A5: Cause specific contributions to the change in female lifespan disparity $e^{\dagger}$, 1994-2000

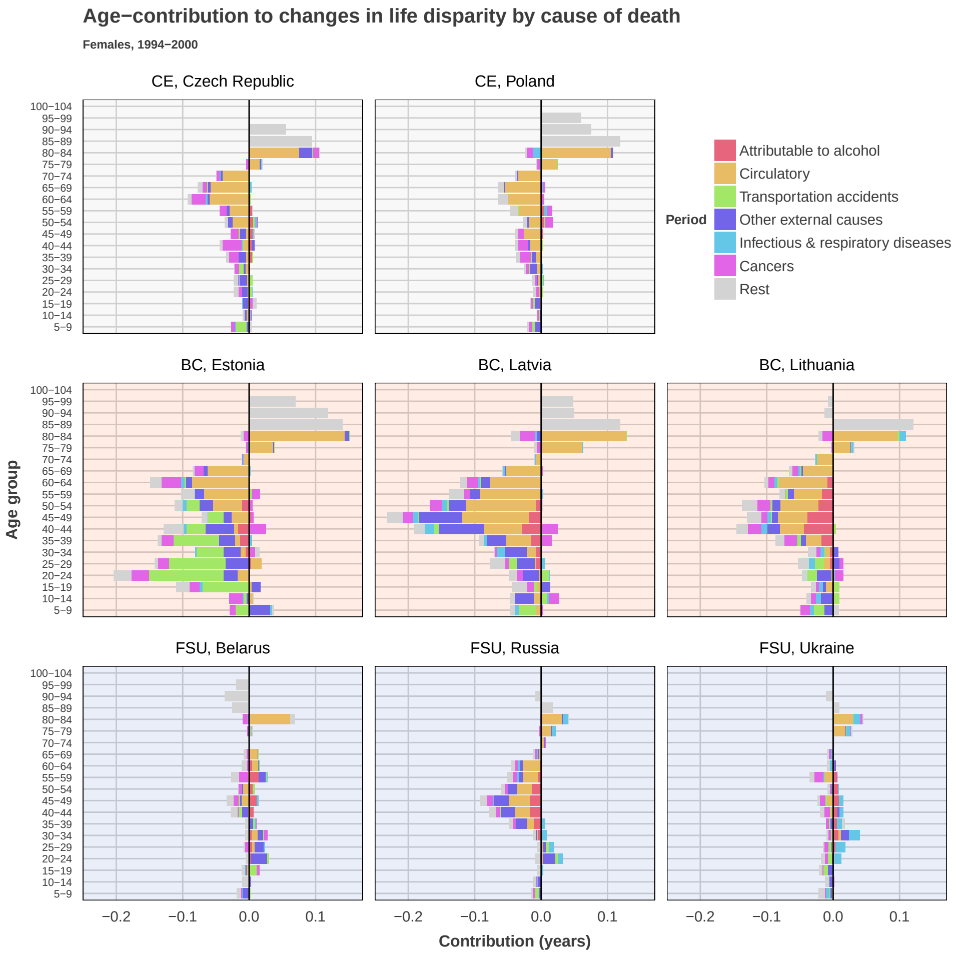

Source: own calculations based on Human Mortality Database (2016) data.

Figure A6: Cause specific contributions to the change in female lifespan disparity $e^{\dagger}$, 2000-2010

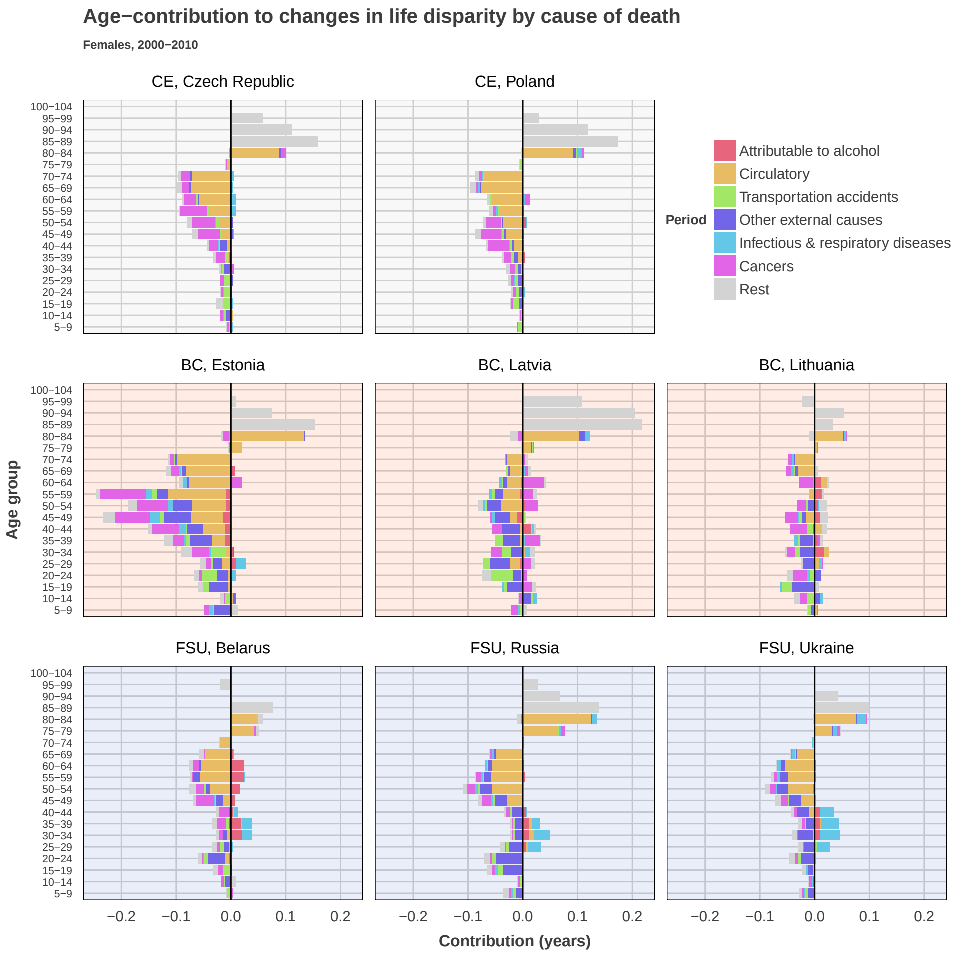

Source: own calculations based on Human Mortality Database (2016) data. Note: data for Poland ends in 2009.

Infant contributions to changes in $e^{\dagger}$ and e_0

Figure A7: Infant contributions, below age 5, to changes in $e^{\dagger}$ and e_0 by period and sex

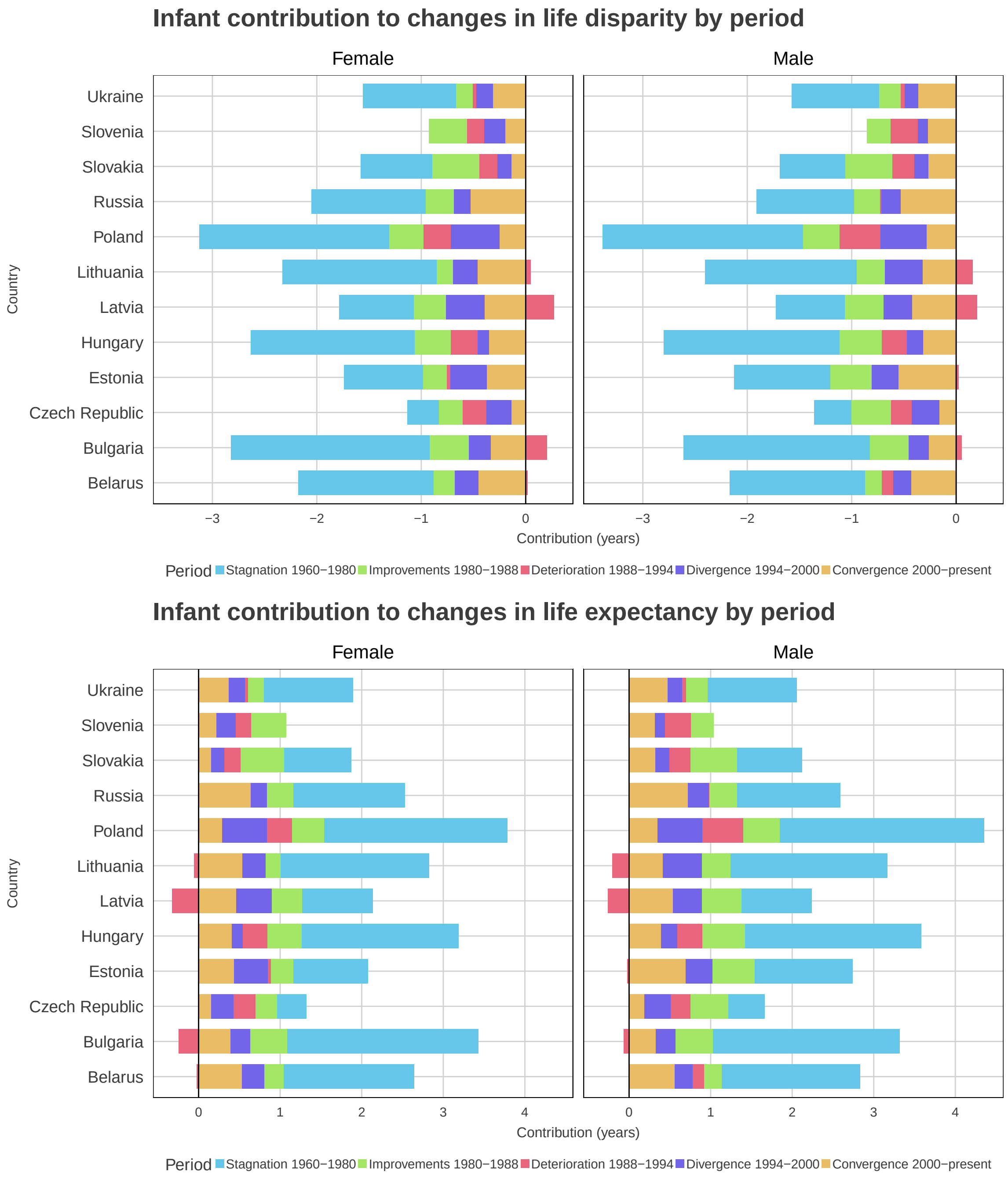

Source: own calculations based on Human Mortality Database (2016) data.

Age and age-cause specific decomposition results for life expectancy

Figure A8: Age-specific contributions to the change in e_0 by periods for males.

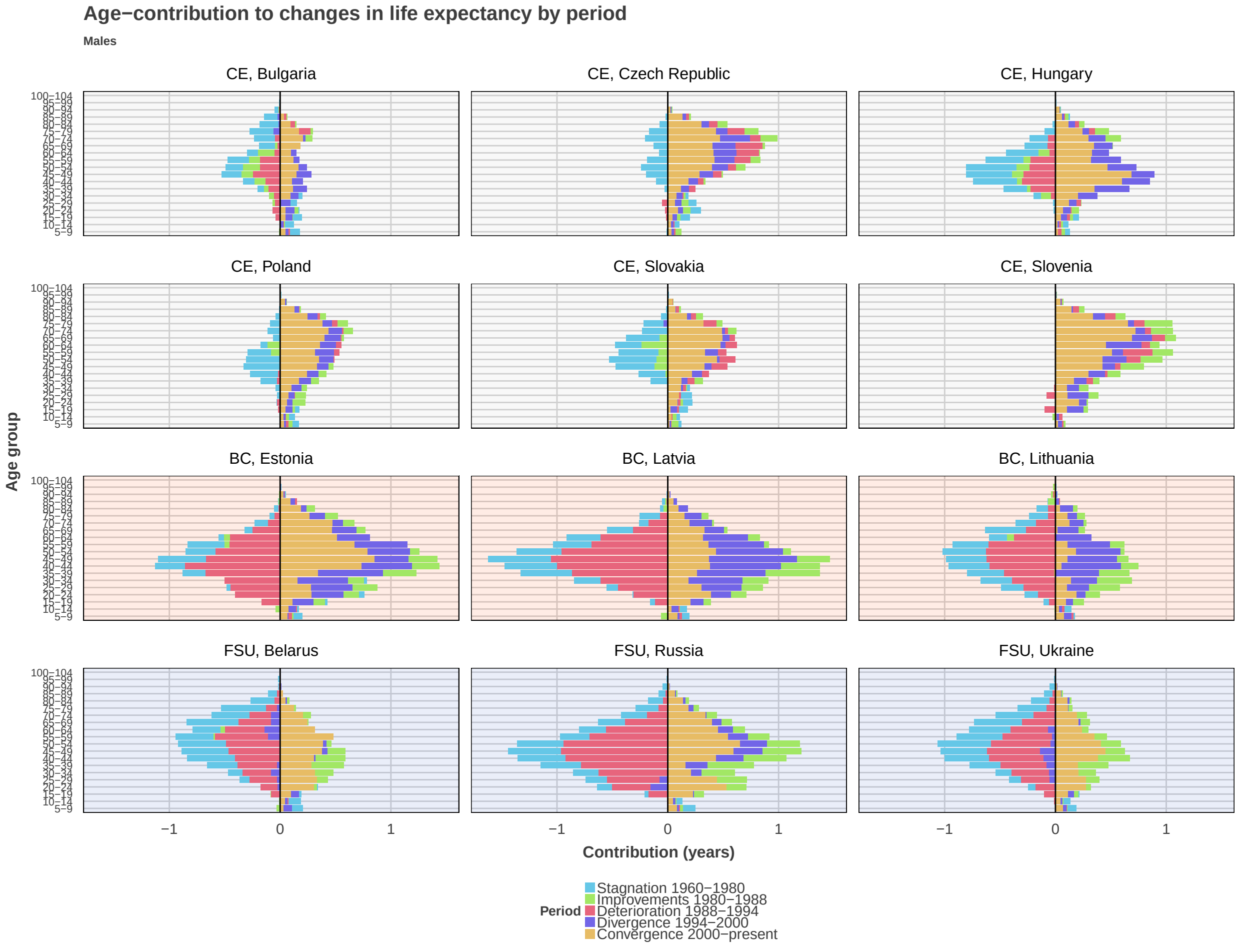

Source: own calculations based on Human Mortality Database (2016) data. Note: data for Slovenia begins in 1983.

Figure A9: Age-specific contributions to the change in e_0 by periods for females.

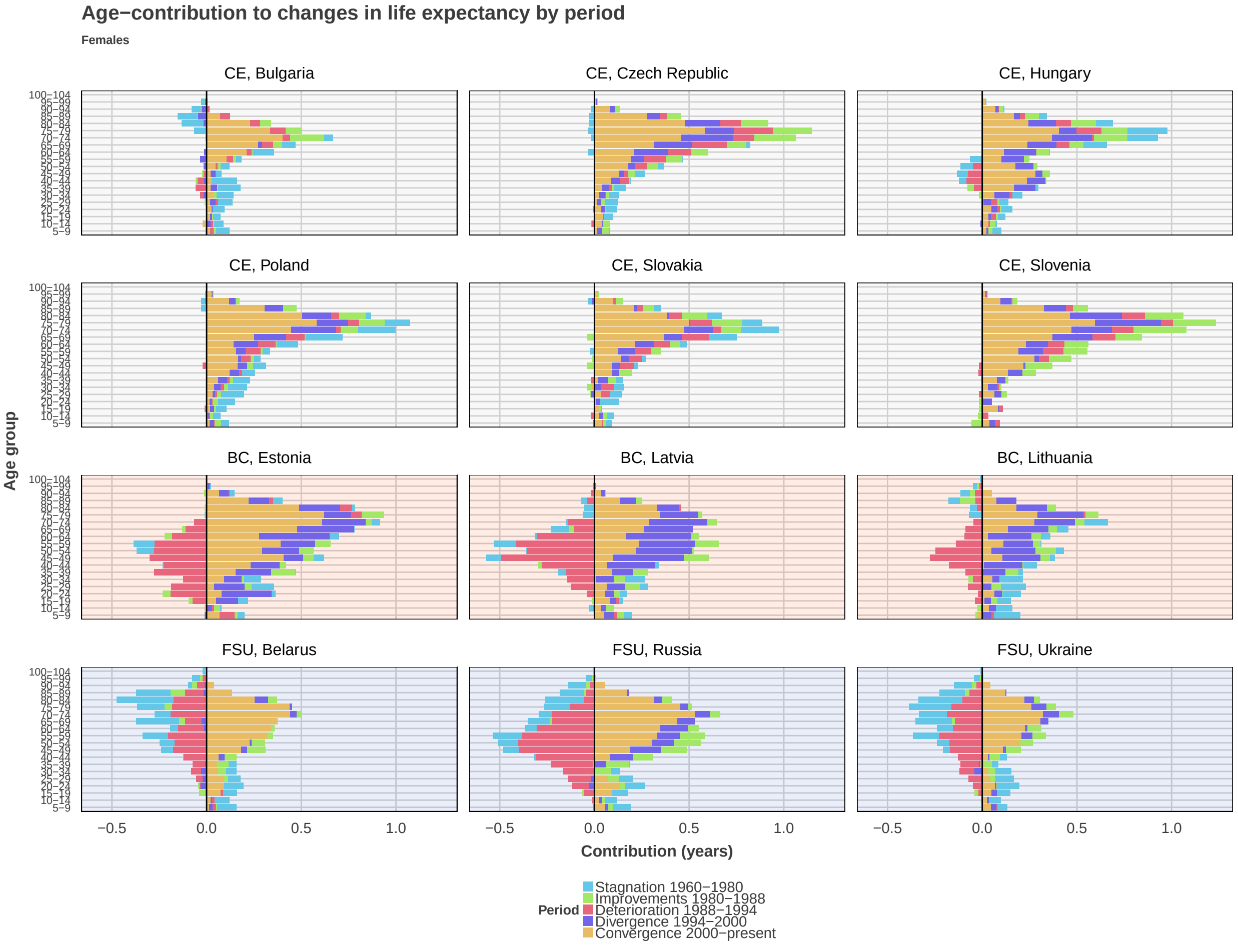

Source: own calculations based on Human Mortality Database (2016) data. Note: data for Slovenia begins in 1983.

Figure A10: Age-cause-specific contributions to the change in e_0 by periods for males 1994-2000.

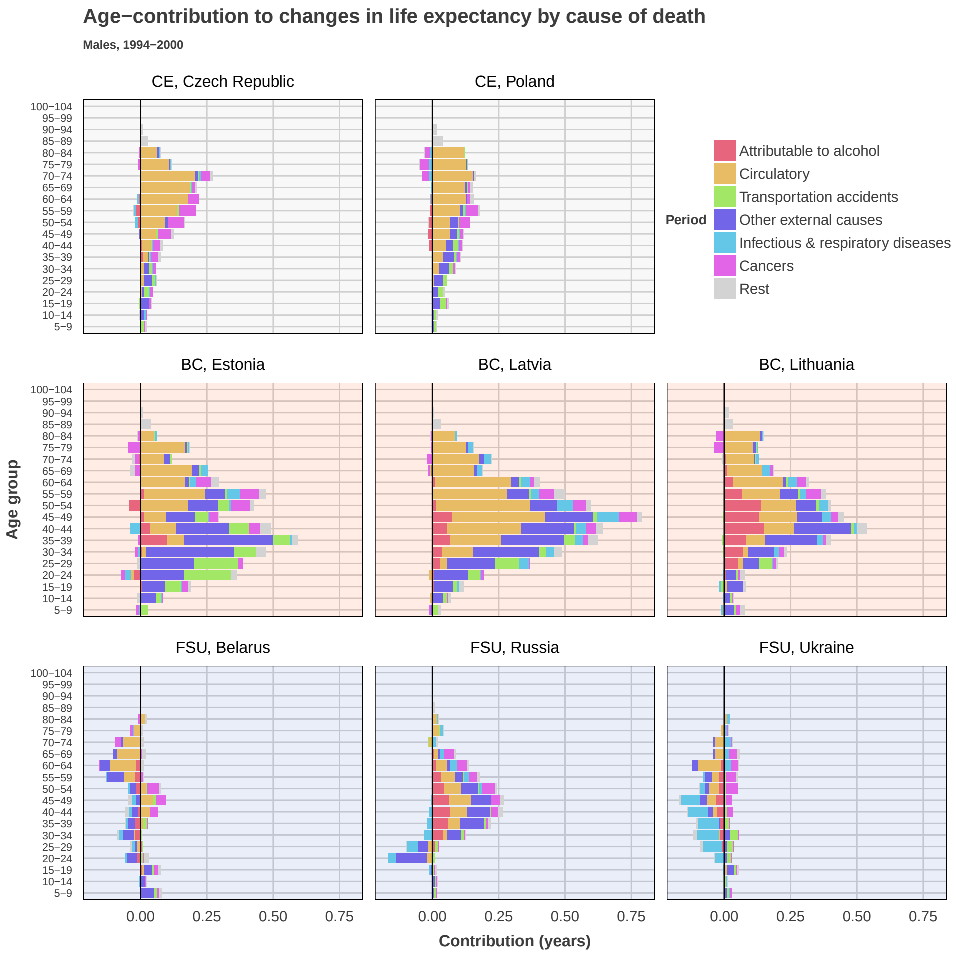

Source: own calculations based on Human Mortality Database (2016) data. Note: data for Slovenia begins in 1983.

Figure A11: Age-cause-specific contributions to the change in e_0 by periods for males 2000-2010.

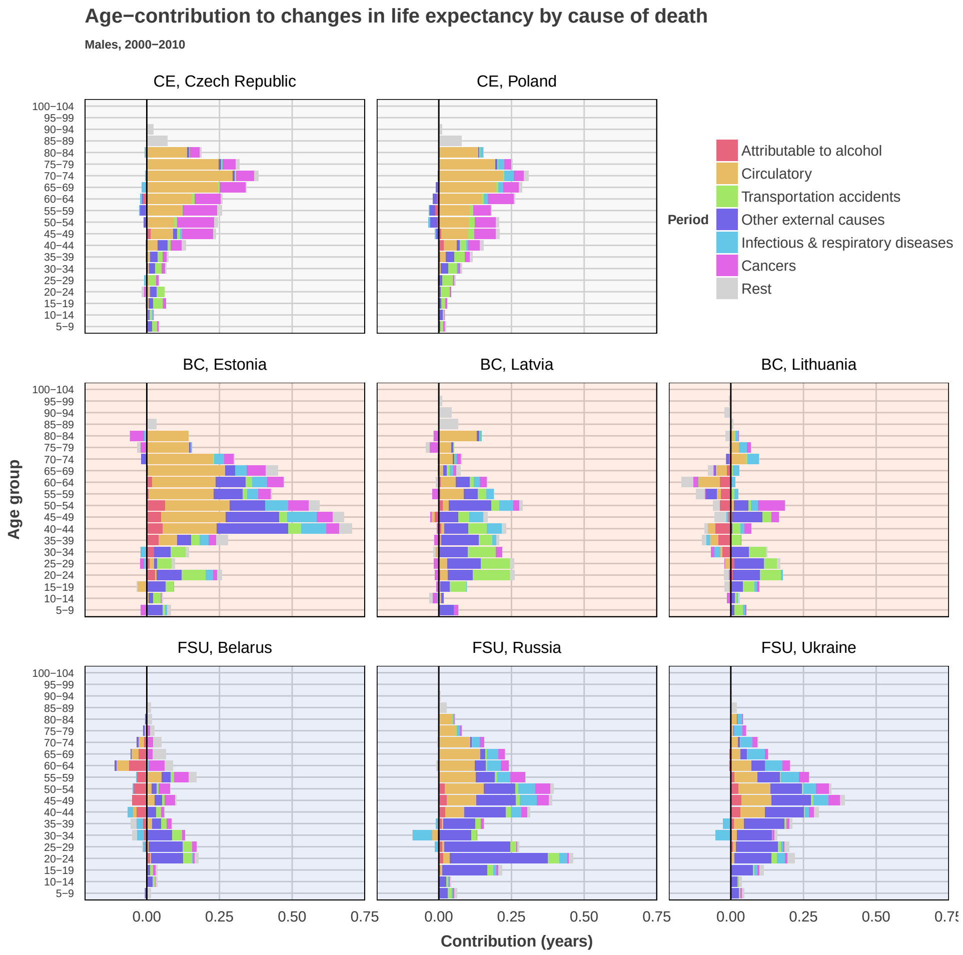

Source: own calculations based on Human Mortality Database (2016) data. Note: data for Slovenia begins in 1983.

Figure A12: Age-cause-specific contributions to the change in e_0 by periods for females 1994-2000.

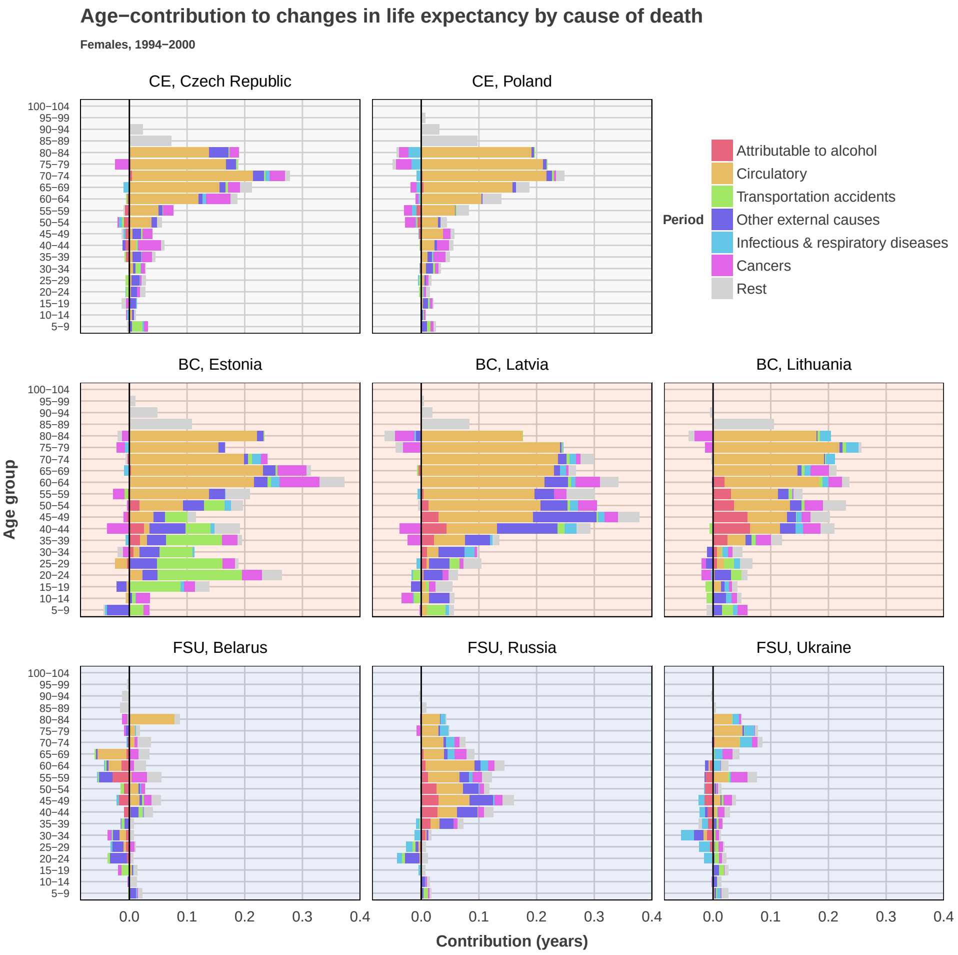

Source: own calculations based on Human Mortality Database (2016) data. Note: data for Slovenia begins in 1983.

Figure A13: Age-cause-specific contributions to the change in e_0 by periods for females 2000-2010.

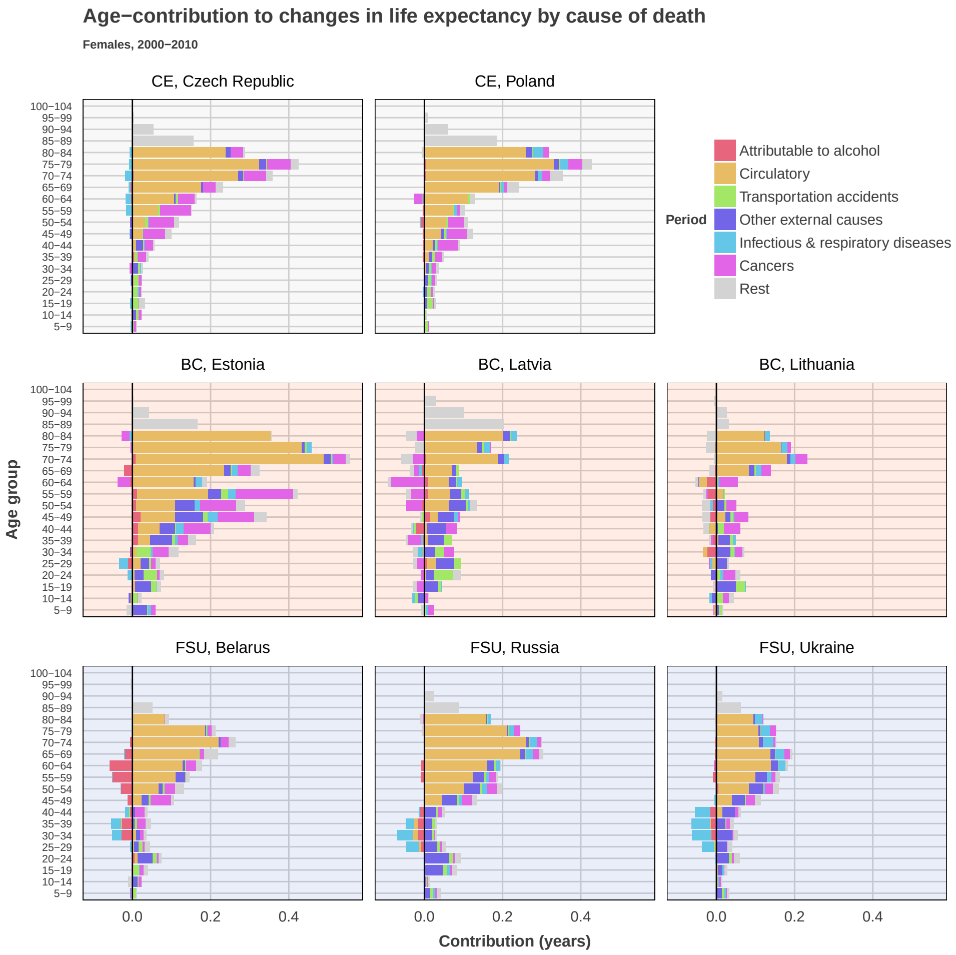

Source: own calculations based on Human Mortality Database (2016) data. Note: data for Slovenia begins in 1983.

Sensitivity analysis without infant mortality and doubling infant mortality

Conditional on surviving to age 5

Figure 14 below shows life expectancy at age 5 versus life expectancy at birth for the Eastern European countries selected in our study. Although the levels differ, major trends are like the ones we show in the paper. For instance, Russia, Latvia and Estonia also show the lower values of life expectancy at age 5, and Slovenia and Czech Republic are the frontrunners in the region after 1990 for males, even without accounting for mortality below age 5.

Similarly, Figure 15 shows life disparity or lifespan variation conditional on surviving to age 5 and at birth. As in the previous figure, the trends are similar to lifespan variation over the full age span. Figure 16 shows the association between changes in life expectancy and lifespan variation conditional on surviving to age 5 and. The results do not change substantively compared to those including mortality under age 5.

We further performed the age-specific decomposition of lifespan variation conditional on surviving to age 5 following the same methodology that we used in the paper. Results are shown in Figure 17 below. The results are very close as if we decompose lifespan variation from age 0 (Figure 4 in the paper).

Figure A14: Life expectancy at age 5 and life expectancy at birth for males.

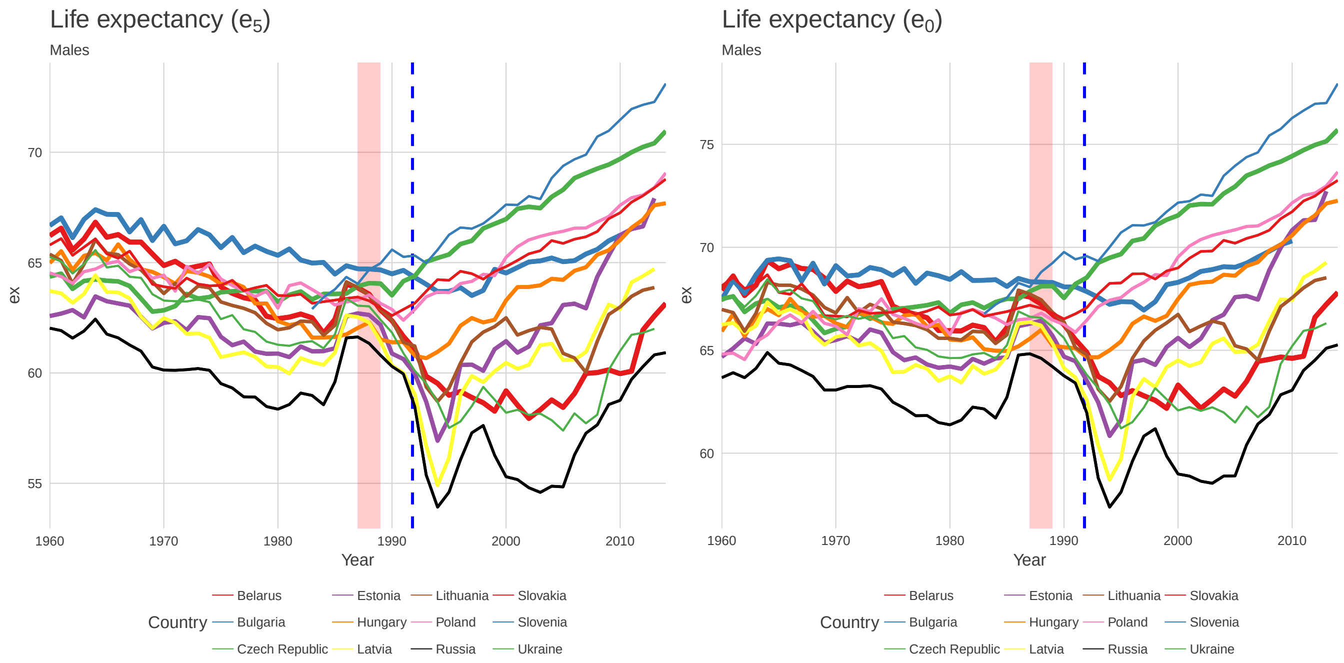

Source: own calculations based on Human Mortality Database (2016) data. Note: data for Slovenia begins in 1983.

Figure A15: Lifespan variation at age 5 and at birth for males.

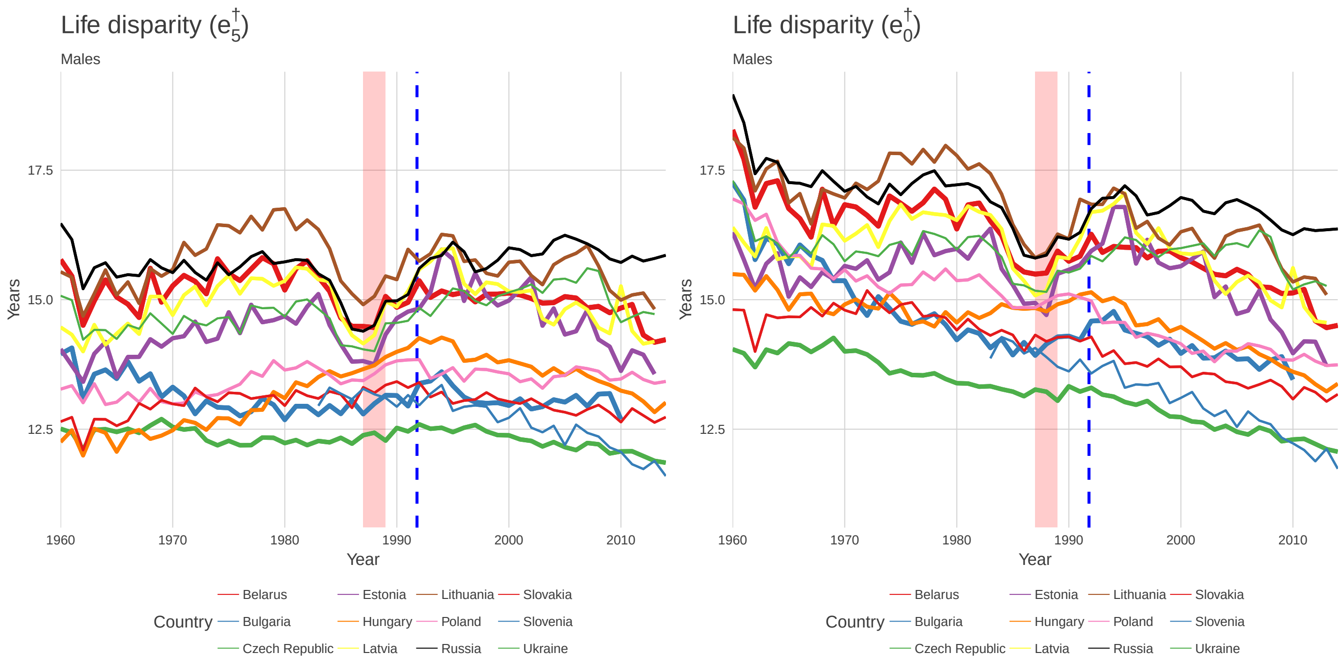

Source: own calculations based on Human Mortality Database (2016) data. Note: data for Slovenia begins in 1983.

Figure A16: Association between changes in life expectancy at age 5 and lifespan variation conditional on surviving to age 5.

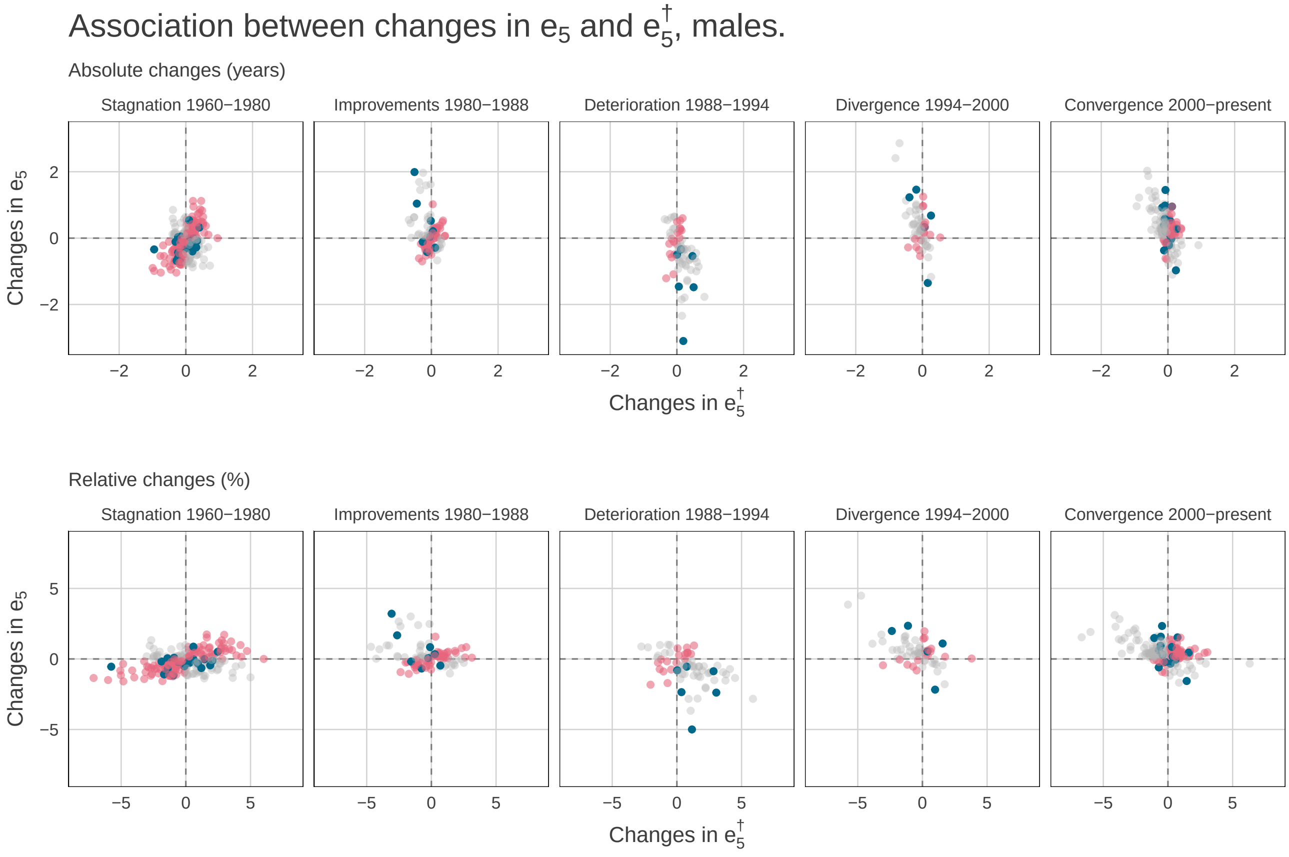

Source: own calculations based on Human Mortality Database (2016) data. Note: data for Slovenia begins in 1983.

Figure A17: Age specific decomposition for life expectancy and lifespan variation at age 5

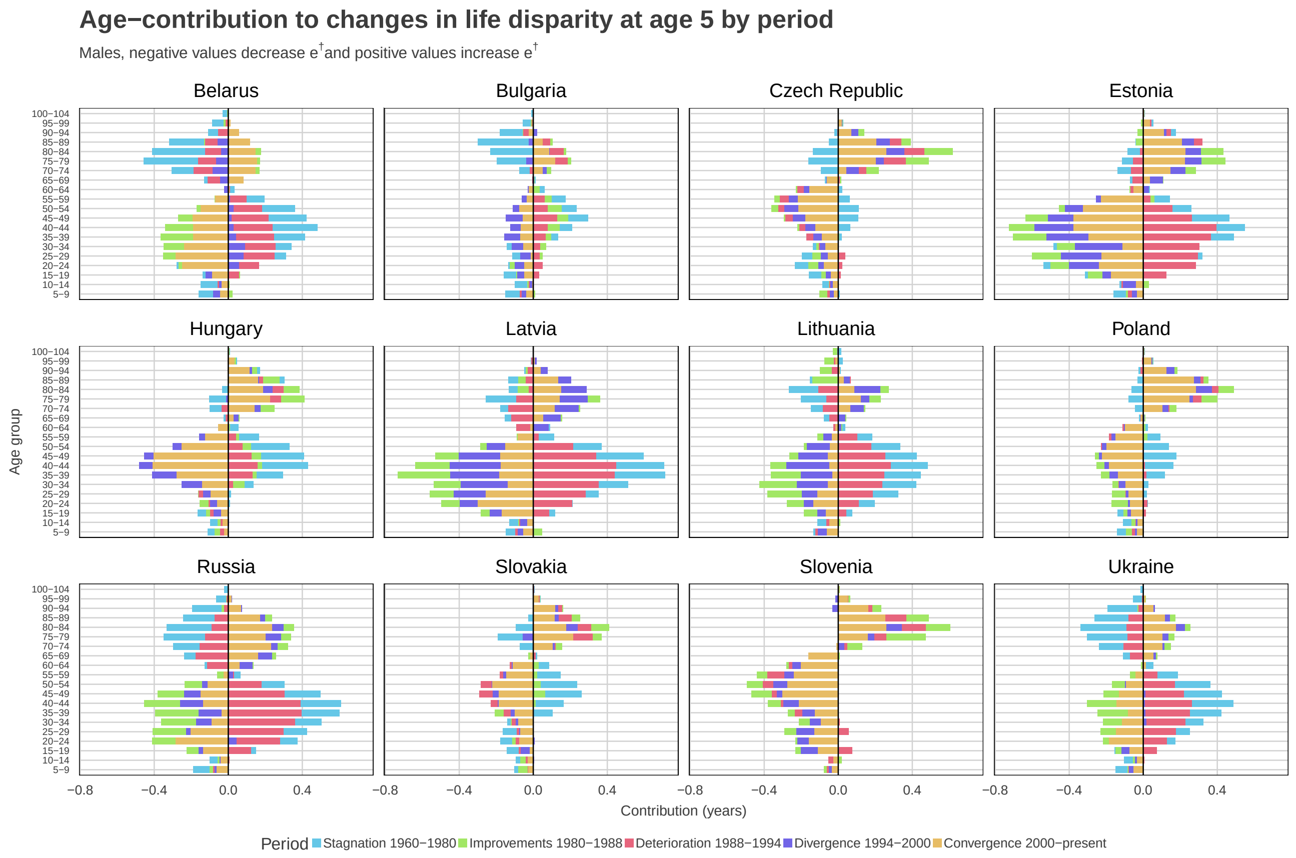

Source: own calculations based on Human Mortality Database (2016) data. Note: data for Slovenia begins in 1983.

Doubling infant mortality

To see the robustness check for different infant mortality scenarios follow https://demographs.shinyapps.io/CEE_App/

Sensitivity analysis with the Gini coefficient

Figure A18: Trends in life expectancy and Gini coefficient by sex for Eastern European countries

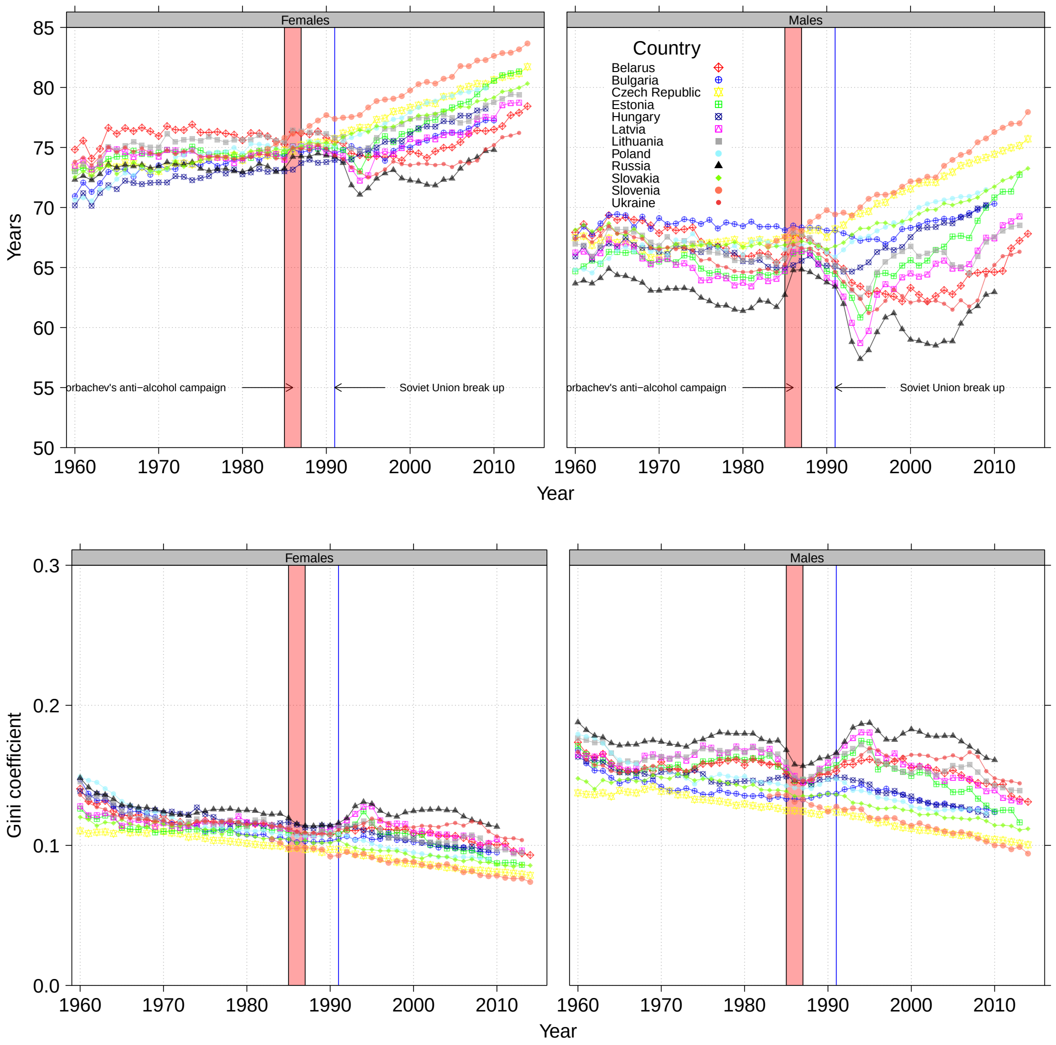

Figure A19: Absolute changes in life expectancy and Gini coefficient by sex for Eastern European countries

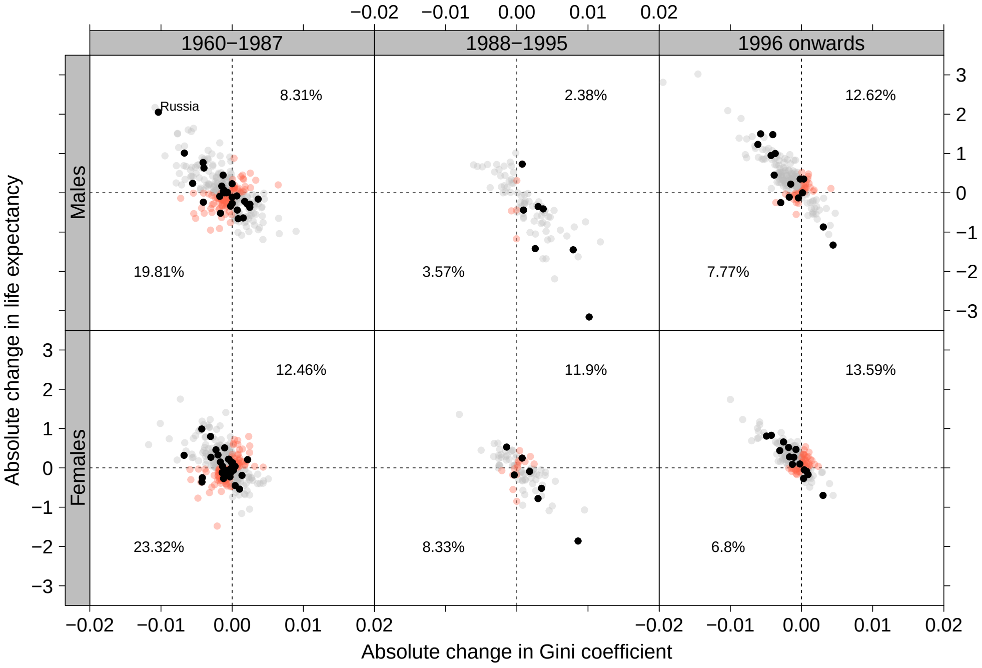

Figure A20: Relative changes in life expectancy and Gini coefficient by sex for Eastern European countries

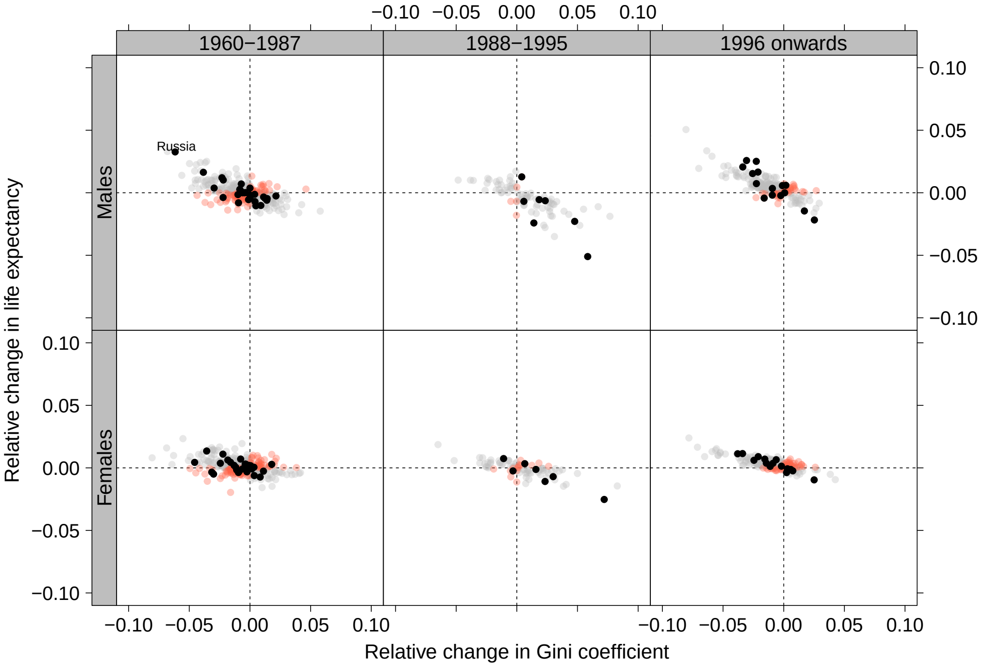

Figure A21: Contributions to changes in Gini coefficient by period

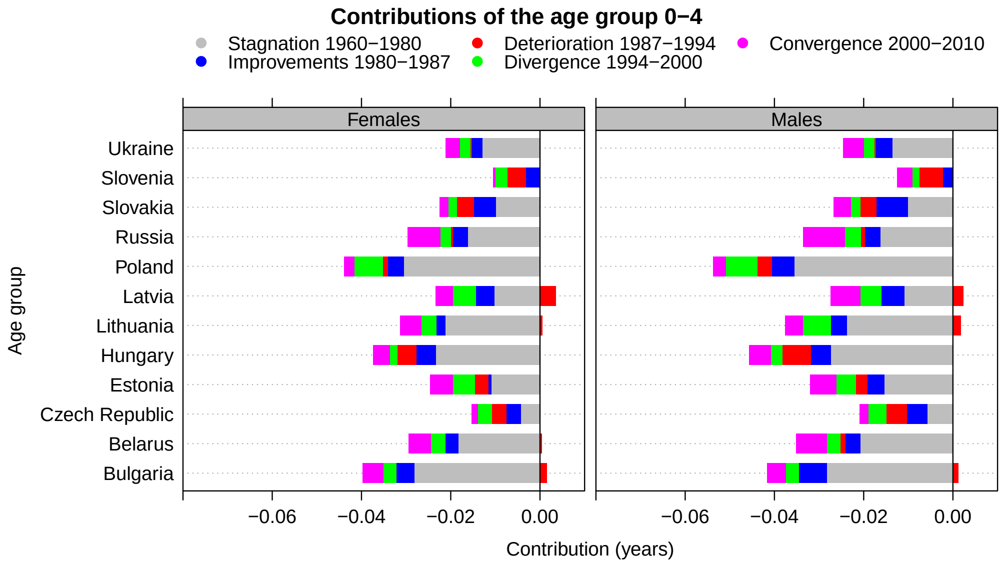

Figure A22: Contributions to changes in Gini coefficient by period, Males.

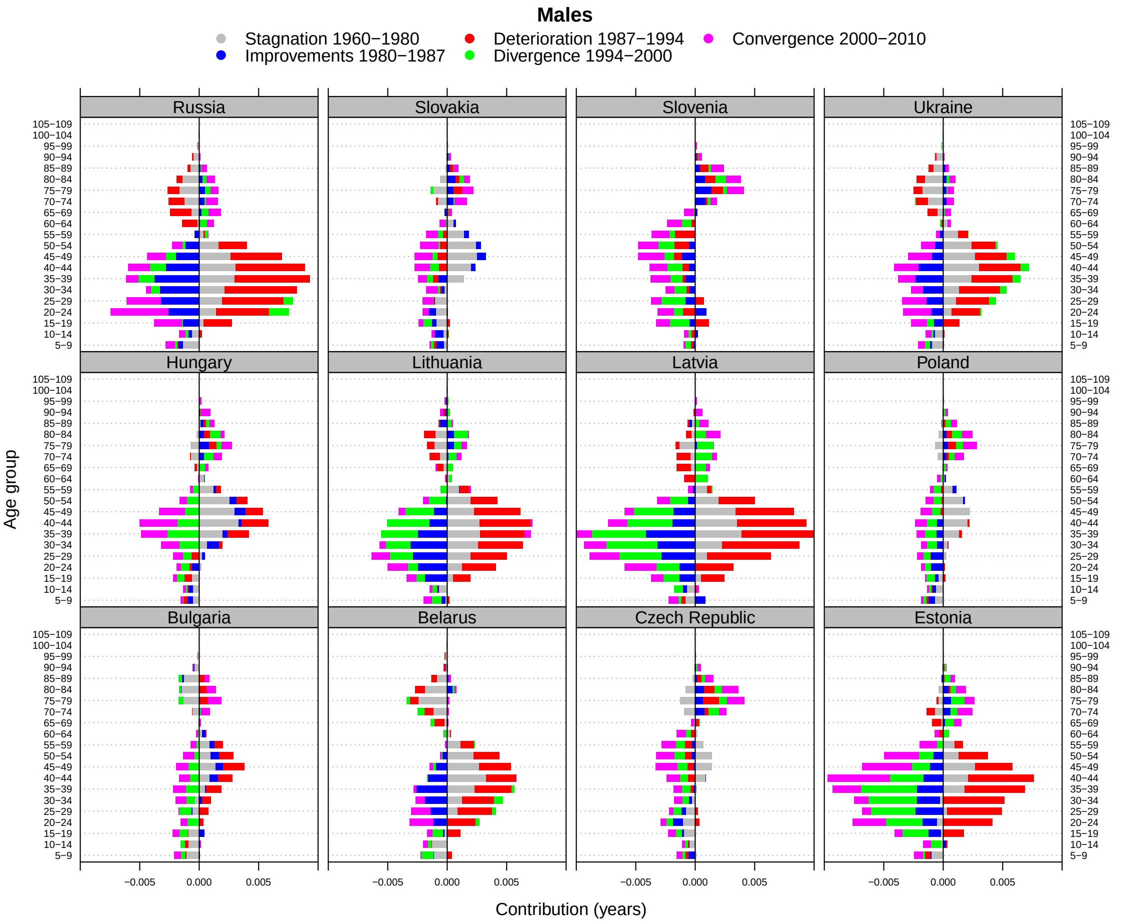

References

Human Mortality Database (2016). University of California, Berkeley (USA), and Max Planck Institute for Demographic Research (Germany).
